# Gβ Regulates Coupling between Actin Oscillators for Cell Polarity and Directional Migration

**DOI:** 10.1371/journal.pbio.1002381

**Published:** 2016-02-18

**Authors:** Oliver Hoeller, Jared E. Toettcher, Huaqing Cai, Yaohui Sun, Chuan-Hsiang Huang, Mariel Freyre, Min Zhao, Peter N. Devreotes, Orion D. Weiner

**Affiliations:** 1 Cardiovascular Research Institute and Department of Biochemistry and Biophysics, University of California San Francisco, San Francisco, California, United States of America; 2 Department of Cell Biology, Johns Hopkins School of Medicine, Baltimore, Maryland, United States of America; 3 Institute for Regenerative Cures, Department of Dermatology, University of California Davis School of Medicine, Sacramento, California, United States of America; 4 Swarthmore College, Swarthmore, Pennsylvania, United States of America; Institute for Systems Biology, UNITED STATES

## Abstract

For directional movement, eukaryotic cells depend on the proper organization of their actin cytoskeleton. This engine of motility is made up of highly dynamic nonequilibrium actin structures such as flashes, oscillations, and traveling waves. In *Dictyostelium*, oscillatory actin foci interact with signals such as Ras and phosphatidylinositol 3,4,5-trisphosphate (PIP_3_) to form protrusions. However, how signaling cues tame actin dynamics to produce a pseudopod and guide cellular motility is a critical open question in eukaryotic chemotaxis. Here, we demonstrate that the strength of coupling between individual actin oscillators controls cell polarization and directional movement. We implement an inducible sequestration system to inactivate the heterotrimeric G protein subunit Gβ and find that this acute perturbation triggers persistent, high-amplitude cortical oscillations of F-actin. Actin oscillators that are normally weakly coupled to one another in wild-type cells become strongly synchronized following acute inactivation of Gβ. This global coupling impairs sensing of internal cues during spontaneous polarization and sensing of external cues during directional motility. A simple mathematical model of coupled actin oscillators reveals the importance of appropriate coupling strength for chemotaxis: moderate coupling can increase sensitivity to noisy inputs. Taken together, our data suggest that Gβ regulates the strength of coupling between actin oscillators for efficient polarity and directional migration. As these observations are only possible following acute inhibition of Gβ and are masked by slow compensation in genetic knockouts, our work also shows that acute loss-of-function approaches can complement and extend the reach of classical genetics in *Dictyostelium* and likely other systems as well.

## Abstract 2

Coupling of individual oscillators regulates biological functions ranging from crickets chirping in unison to the coordination of pacemaker cells of the heart. This study finds that a similar concept—coupling between actin oscillators—is at work within single slime mold cells to establish polarity and guide their direction of migration.

## Author Summary

The actin cytoskeleton of motile cells is comprised of highly dynamic structures. Recently, small oscillating actin foci have been discovered around the periphery of *Dictyostelium* cells. These oscillators are thought to enable pseudopod formation, but how their dynamics are regulated for this is unknown. Here, we demonstrate that the strength of coupling between individual actin oscillators controls cell polarization and directional movement. Actin oscillators are weakly coupled to one another in wild-type cells, but they become strongly synchronized after acute inactivation of the signaling protein Gβ. This global coupling impairs sensing of internal cues during spontaneous polarization and sensing of external cues during directional motility. Supported by a mathematical model, our data suggest that wild-type cells are tuned to an optimal coupling strength for patterning by upstream cues. These observations are only possible following acute inhibition of Gβ, which highlights the value of revisiting classical mutants with acute loss-of-function perturbations.

## Introduction

For cells to move, their cytoskeletal structures become spatially organized by internal polarity signals [1–3] as well as external chemoattractant [4–6]. How such signaling cues tame actin dynamics to produce a pseudopod and guide cellular motility remains a key question in eukaryotic chemotaxis.

By now, several key regulators of the actin cytoskeleton have been identified: in most cells, nucleation promoting factors (NPFs) of the Wiskott-Aldrich Syndrome Protein (WASP) and SCAR/WAVE family stimulate actin nucleation through the Arp2/3 complex and are essential for regulating polarity and motility for cells ranging from *Dictyostelium* [6,7] to metazoans [8–10]. NPFs themselves are regulated by self-association on the plasma membrane [1,11] and actin polymerization-based autoinhibition [1,12,13]; the actin polymer that they generate facilitates the removal of these NPFs from the plasma membrane. These positive and negative feedback interactions of the NPFs [1,14] and other actin regulators give rise to a range of highly dynamic, free-roaming, nonequilibrium actin structures such as flashes and traveling waves [1,2,5,6,15–21], but how the actin machinery is coaxed to form these very different activity patterns is not well understood.

Particularly striking displays of NPF and actin dynamics are actin oscillations, which can be observed in many cell types and contexts [1,2,5,22,23]. Biological oscillations are typically generated through a combination of (1) fast positive feedback, which amplifies small signals into an all-or-none output; and (2) delayed inhibition, which turns the output off and resets the system for the next pulse. By spatially coupling oscillators, spreading or synchronization over long distances can be achieved [24–26].

Recently, small oscillating SCAR/WAVE foci have been discovered at the periphery of *Dictyostelium* cells [2]. These foci may constitute the basic cytoskeletal units from which pseudopods are formed. In the absence of signaling cues, these oscillators are present but lead to only small undulations of the cell boundary. In response to upstream signals, however, full-blown protrusions emerge [2,27–31], likely from the coordination of these foci. Some intracellular signals (such as Ras and phosphatidylinositol 3,4,5-trisphosphate [PIP_3_]) have been identified that affect this transition, but whether other signals link receptor activation with the SCAR/WAVE foci, and, more generally, which properties of the foci are modulated to enable large-scale coordination, are not known.

Here, we find that the heterotrimeric G-protein subunit Gβ sets the coupling range of an actin-based activator—inhibitor system. Specifically, acute sequestration of Gβ leads to strong global synchronization of normally weakly coupled cytoskeletal oscillators, and these effects are independent of known upstream regulators of these oscillators, such as Ras and PIP_3_. We show that this extended range of spatial coupling is detrimental for cell polarity, cell motility, and directional migration. To guide our intuition for how coupling between oscillators could affect the cell’s ability to sense directional cues, we developed a simple mathematical model that represents its minimal features. Simulations show that the ability to sense a noisy input signal is facilitated by an intermediate strength of oscillator coupling, allowing different membrane regions to share information about the stimulus. We propose that in wild-type cells, Gβ sets the coupling strength of actin oscillators to an appropriate level to sense directional upstream cues.

## Results

### Engineering Rapamycin-Based Acute Inactivation of Gβ

Strong loss-of-function phenotypes in cell motility are rare [6,32–38]. One reason may be that genetic perturbations are slow to act and may give cells time to compensate for gene loss [39–42]. Redundantly controlled processes like actin rearrangements during motility may be particularly susceptible to such compensation. To overcome this limitation, we developed a system that enables fast loss-of-function perturbations to cell signaling events involved in *Dictyostelium* cell motility. Here, we focus on its application to Gβ.

Heterotrimeric G-proteins consist of one α, β, and γ subunit and link receptor-mediated signals to directed migration and polarization in eukaryotic cells ranging from yeast to neutrophils to *Dictyostelium* [43–46]. Both intra- and extracellular signals can regulate the cytoskeleton, yet while knockout of the sole Gβ protein in *Dictyostelium* completely blocks chemotaxis, basal cytoskeletal dynamics and other directional responses such as shear-flow-induced motility and electrotaxis are still present, although somewhat reduced [2,3,44,47,48].

**Fig 1:**
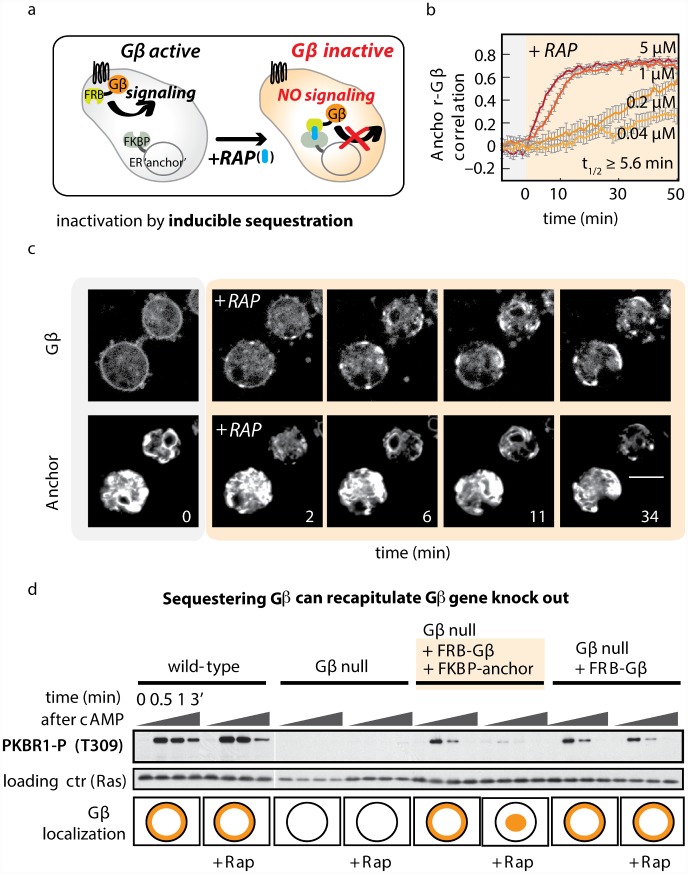
Inducible protein sequestration as a method to acutely inactivate Gβ. (A) Inducible sequestration can be exploited to inactivate a protein of interest. Using the small molecule rapamycin (RAP), FRB-tagged Gβ can be recruited to an FKBP-tagged “anchor” at the endoplasmic reticulum (ER). Addition of RAP sequesters Gβ from its normal site of action at the plasma membrane and prevents it from activating downstream effectors. (B) Timecourse of Gβ sequestration. In cells lacking endogenous Gβ, but expressing FRB-RFP-Gβ and calnexinA-YFP-FKBP as an anchor at the ER, the speed and extent of sequestration were assayed by measuring the spatial correlation between YFP and RFP signals. For the highest dose of RAP, half-maximal heterodimerization is achieved within 5.6 min. To keep cells immobile, the experiment was performed in the presence of 10 μM latrunculinA. The spatial correlations between fluorescence signals from Gβ and anchor are plotted (n ≥ 20 cells per condition; mean +/- standard error of the mean [SEM]). Raw data can be found in S1 Data. (C) Representative images from a Gβ sequestration timecourse described in (B). Scale bar = 10 μm. (D) Gβ sequestration recapitulates Gβ-null phenotypes for receptor-stimulated signaling. Timecourses of chemoattractant stimulation (cyclic-AMP [cAMP]; 10 μM) are shown in four strains: wild-type (wt), Gβ-null, and cells expressing one or both components of the Gβ sequestration system. Each strain was stimulated in the presence and absence of rapamycin (5 μM; > 20 min incubation). Blot shows phosphorylation of PKBR1 (T309); Ras is used as a loading control. Schematic indicates the localization of Gβ (in orange) for each condition in test strains and the published localization for wt and Gβ-null cells. Further examples of signaling events blocked after Gβ sequestration can be found in S1 Fig.

Gβ requires plasma membrane localization in order to signal; thus, removal from the plasma membrane should prevent it from activating downstream effectors. As Gβ is continually exchanged between membrane and cytoplasm with a half-life of 5 s [49], it should be possible to trap it by association with an internal anchor. We built a Gβ sequestration system using a chemical dimerization approach whereby the association of two protein domains (FKBP and FRB) is induced by the small molecule rapamycin [50–54]. Starting with Gβ-null cells [44], we expressed an FRB—Gβ fusion protein and an endoplasmic reticulum (ER)-localized FKBP (FKBP-calnexinA [55]). Thus, addition of rapamycin should drive Gβ relocalization to the ER and suppress its signaling function, effectively rendering cells Gβ-null in an acute fashion (Fig 1A).

To test for rapamycin-induced sequestration, we measured the extent of ER-localized Gβ in single cells over time following rapamycin addition. We computed the correlation between each cell’s fluorescence intensity in the ER anchor and Gβ channels to assess co-localization. FRB-RFP-Gβ was rapidly sequestered from the plasma membrane and increasingly co-localized in large clusters with FKBP-YFP-calnexinA (S1 Movie). Sequestration is fast: half-maximal correlation occurred 5.6 min after addition of the highest dose (5 μM) of rapamycin that was tolerated by cells (Fig 1B and 1C, S1 Data). Sequestration kinetics were similar for both 5 μM rapamycin and 1 μM rapamycin. Therefore, unless indicated otherwise, we used the lower concentration for subsequent experiments.

Gβ-null cells fail to transmit many signals triggered by G-protein-coupled receptors (GPCRs) [44,56–59], and we should be able to recapitulate these defects with our sequestration approach. We thus assayed whether relocalization of Gβ to the ER inhibits transmission of signals from GPCRs to downstream effectors. Stimulating wild-type cells with chemoattractant (cAMP) triggers transient responses, including phosphorylation of PKBR1, and this response is abolished in Gβ-null cells [33,56]. We found that introducing our FRB-Gβ construct in Gβ-null cells rescued the PKBR1 response. Acute sequestration of FRB-Gβ to the ER anchor blocked PKBR1 phosphorylation, but only when all three components of our system—the ER anchor, FRB-Gβ, and rapamycin—are present (Fig 1D). Unfortunately experiments using the inducible sequestration system in developed cells were often problematic: Tagged Gβ and anchor components were frequently degraded during starvation and, likely as a consequence, cells failed to complete their developmental cycle. However, this problem was not observed in vegetative cells, in which the sequestration components remained intact. Gβ-dependent, chemoattractant-stimulated responses in vegetative cells, such as Ras activity and PIP_3_ production [57,59,60], could also be blocked by Gβ-sequestration (S1 Fig). Taken together, these results demonstrate that in the absence of rapamycin, our inducible sequestration system sustains key Gβ-dependent signaling events. In the presence of rapamycin, Gβ is sequestered from its site of action, thereby blocking receptor-based signaling. In this respect, sequestration of Gβ recapitulates Gβ-null cells.

### Gβ Sequestration Impairs Directional Migration

To probe for phenotypes that may only be apparent after rapid loss of Gβ, we turned to directional motility assays. We measured the behavior of Gβ-sequestered cells presented with two different directional cues—an attractive chemical (folate) or electric fields—and compared their responses with wild-type and Gβ-null cells. While chemotaxis is strictly dependent on Gβ, electrotaxis, the directed migration of *Dictyostelium* cells in response to electric fields, is not. While Gβ-null cells cannot move up a chemical gradient, they can move down electrical potential [44,47].

We took advantage of the heterogeneity in expression of components in Gβ-sequestered cells to internally control experiments. We can distinguish behavior of cells that, in the presence of rapamycin, are functionally wild-type (expressing RFP-FRB- Gβ, but no CFP-FKBP-anchor), Gβ-null (with no detectable RFP-FRB- Gβ expressed), or Gβ-sequestered (expressing both RFP-FRB- Gβ and CFP-FKBP-anchor). For chemotaxis, we further compared these populations to true wild-type and true Gβ-null cells.

**Fig 2:**
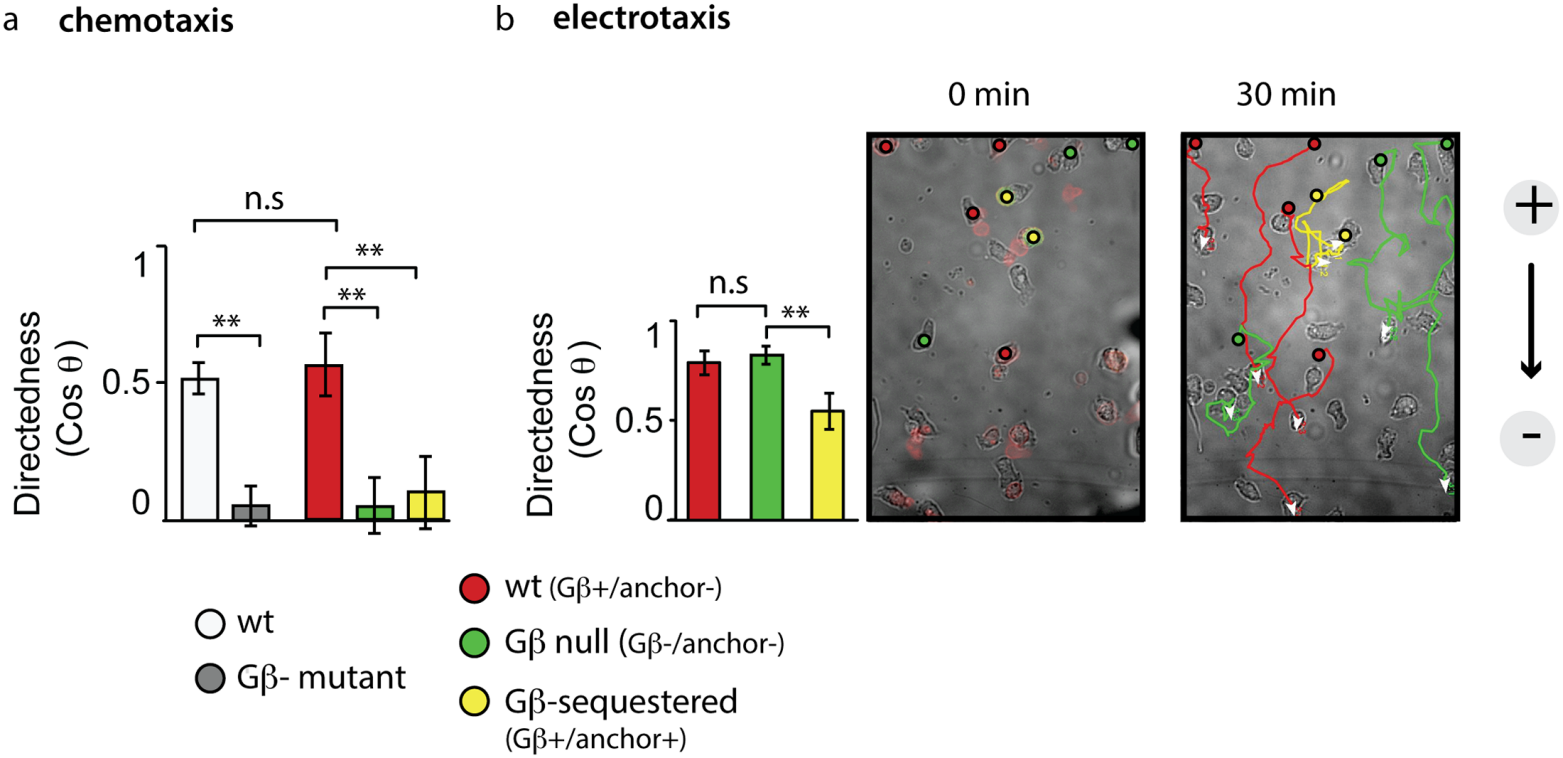
Gβ sequestration impairs directional migration. (A) Cells of the Gβ sequestration strain were incubated with rapamycin and exposed to a gradient of folate. Based on the expression of sequestration components, different subpopulations were identified, and directionality was measured after 30 min of migration. Plotted are the means (+/- S.E.M) of wild-type (wt): Gβ+/anchor- cells (*n* = 30, red); Gβ-null (Gβ-): Gβ-/anchor- cells (*n* = 48, green); and Gβ-sequestered: Gβ+/anchor+ cells (*n* = 31, yellow). ** indicates a highly significant *p*-value of < 0.02; n.s. indicates a not-significant *p*-value of > 0.05 (Student’s two tailed *t* test). Data are derived from five videos in two independent experiments. For comparison, directedness of wt (DH1) and Gβ- cells (*n* = 97 and *n* = 98, data from two videos in single experiments, respectively) is shown in light and dark grey bars. Raw data can be found in S1 Data. (B) Cells of the Gβ sequestration strain were incubated with rapamycin and exposed to an electrical field. Based on the expression of sequestration components, different subpopulations were identified, and directionality was measured after 30 min of migration. Plotted are the means (+/- stdev) of wt: Gβ+/anchor- cells (*n* = 33, red), Gβ-null: Gβ-/anchor- cells (*n* = 34, green); and Gβ-sequestered: Gβ+/anchor+ cells (*n* = 34, yellow). ** indicates a highly significant *p*-value of < 0.01; n.s. indicates a not-significant *p*-value of > 0.05 (Student’s two tailed *t* test). Data are combined from several fields of view of movies recorded on two separate days. A movie corresponding to the stills in Fig 2B is included as S2 Movie. Raw data can be found in S1 Data.

We find that just as unsequestered cells resemble wild-type cells, Gβ-sequestered cells behave similarly to Gβ-null cells in chemical gradients. In the presence of Gβ, cells move directionally, while in the absence of functional Gβ (through sequestration or knockout), directionality is lost (Fig 2A). Furthermore, Gβ-sequestered cells (0.4 +/- 0.1 μm/min; *n* = 31; +/- SEM) as well as true Gβ-null cells (0.4 +/- 0.05 μm/min; *n* = 98; +/- SEM) move at a reduced speed compared to unsequestered (1.1 +/- 0.2 μm/min; *n* = 30; +/- SEM) or true wild-type (2.5 +/- 0.15 μm/min; *n* = 97; +/- SEM) cells.

In contrast, in electrical fields, the behavior of Gβ-sequestered cells differs from the Gβ knockout. Compared to wild-type and Gβ-null cells, Gβ-sequestered cells show a significant decrease in their directionality during electrotaxis (Fig 2B and S2 Movie). Furthermore, the speed of translocation in Gβ-sequestered cells (2.1 +/- 0.22 μm/min, mean +/- SEM; *n* = 34) was reduced compared to wild-type (3.8 +/- 0.23 μm/min, mean +/- SEM; *n* = 34; Student’s two tailed *t* test: *p* < 10^-6^) and Gβ-null cells (2.9 +/- 0.23 μm/min, mean +/- SEM; *n* = 33; Student’s two tailed *t* test: *p* < 0.006).

### Gβ Sequestration Drives Large-Scale Oscillations of Cortical F-actin

**Fig 3:**
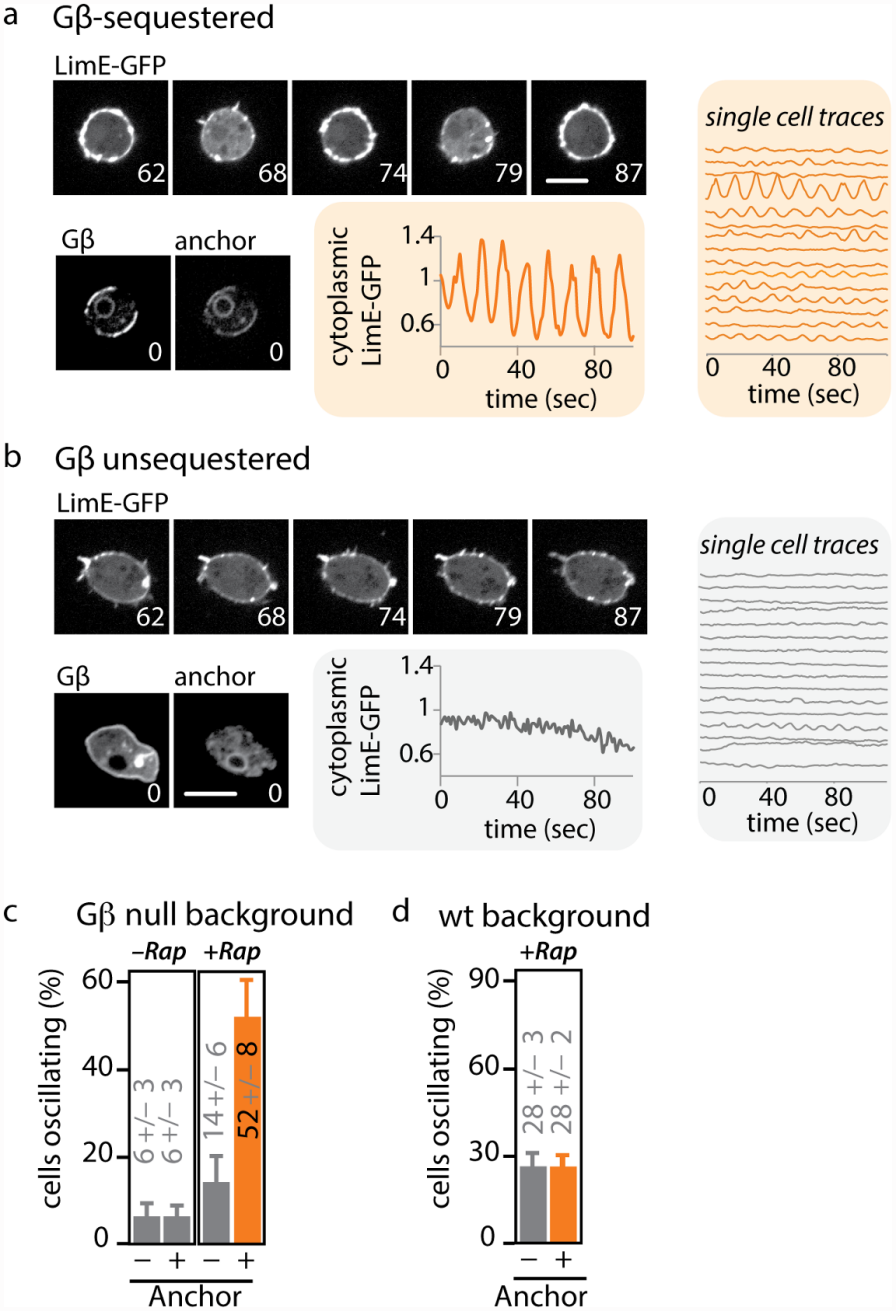
Acute Gβ sequestration leads to oscillations in cortical F-actin. (A) Acute sequestration of Gβ induces cytoplasmic oscillations of the F-actin reporter LimE-GFP. Cells were treated with 1 μM rapamycin, and LimE-GFP (upper panels) was imaged over time. Graphs show cytoplasmic LimE-GFP quantified from individual cells. Lower panels: FRB-RFP-Gβ and calnexinA-YFP-FKBP images show colocalization (sequestration) of Gβ at the anchor. Scale bar = 5 μm. Numbers indicate time in seconds. Corresponding oscillations at the cortex can be seen in S2 Fig. (B) Behavior of the F-actin reporter in Gβ unsequestered cells. LimE-GFP (upper panels) was imaged over time. Graphs show cytoplasmic LimE-GFP quantified from individual cells. Lower panels: FRB-RFP-Gβ and calnexinA-CFP-FKBP images show distinct Gβ and anchor localization. Scale bar = 5 μm. Numbers indicate time in seconds. (C) The percentage of oscillating cells was quantified from cells expressing LimE-GFP and FRB-RFP-Gβ, either in the presence (+ anchor) or absence (–anchor) of calnexinA-YFP-FKBP. In both strains, cells were left untreated (–Rap) or incubated with 1 μM rapamycin (+Rap) for at least 20 min (*n* ≥ 150 cells per condition from three independent days; plotted are means +/- SEM). Raw data can be found in S1 Data. (D) The oscillatory phenotype is rescued by performing Gβ sequestration in the presence of wild-type Gβ. This indicates that sequestration of Gβ induces a loss-of-function phenotype. Wild-type cells expressing FRB-RFP-Gβ were incubated with 1 μM rapamycin (+Rap) for at least 20 min. LimE-GFP oscillations were compared between cells that co-expressed the anchor (calnexinA-CFP-FKBP) or lacked the anchor. (- anchor: *n* = 91; mean +/- stdev; + anchor: *n* = 23; mean +/- stdev). Further experiments presented as supplement: Oscillations of LimE are due to loss, and not gain, of Gβ function (S3 Fig). The computational pipeline used to analyze oscillations is presented in S4 Fig. In some cases, Gβ sequestration also induces waves of actin polymerization that travel around the cell perimeter (S5 Fig). Oscillations of LimE start rapidly after Gβ is sequestered (S6 Fig). Raw data can be found in S1 Data.

Closer examination of Gβ-sequestered cells by confocal microscopy revealed a striking change in the organization of the actin cytoskeleton. While wild-type cells have fairly stable levels of cortical and cytoplasmic actin, sequestration of Gβ induces striking oscillations of LimE-GFP, a reporter for dynamic F-actin (Fig 3A and 3B) [61]. Periodic loss of cytoplasmic LimE-GFP intensity is accompanied by a corresponding accumulation of F-actin around the entire periphery of the cell (S2 and S3 Figs). The cytoskeletal oscillations induced by Gβ sequestration are present in the majority of cells and have well-defined characteristics. By automatically tracking cells over time and measuring their cytoplasmic LimE-GFP intensity, we identified oscillating cells from the characteristic peak induced in their Fourier spectrum (S4 Fig). After rapamycin addition, the fraction of oscillating cells rises from 6% to 52%, but only when the ER anchor is co-expressed (Fig 3C and S1 Data). The period of oscillation (measured as the peak frequency of the Fourier-transformed signal) is tightly controlled across all oscillating Gβ-sequestered cells (12.9 +/- 3.2 s, *n* = 83) (S4 Fig). We also observed a second F-actin phenotype upon acute loss of Gβ. In ~10% of cells, waves of F-actin polymerization travel around the cell perimeter with a similar period as the whole field oscillations, taking 10–20 s for a full cycle (S5 Fig and S3 Movie).

Two lines of evidence confirm that acute Gβ loss of function through sequestration is required to initiate this actin oscillation phenotype. First, oscillations are not observed when the ER is forced into proximity of the plasma membrane, arguing against an ER-specific recruitment phenotype (S3 Fig). Most importantly, when Gβ is overexpressed and sequestered in wild-type cells (which harbor endogenous Gβ that cannot be recruited), no actin oscillations are induced (Fig 3D and S1 Data).

**Fig 4:**
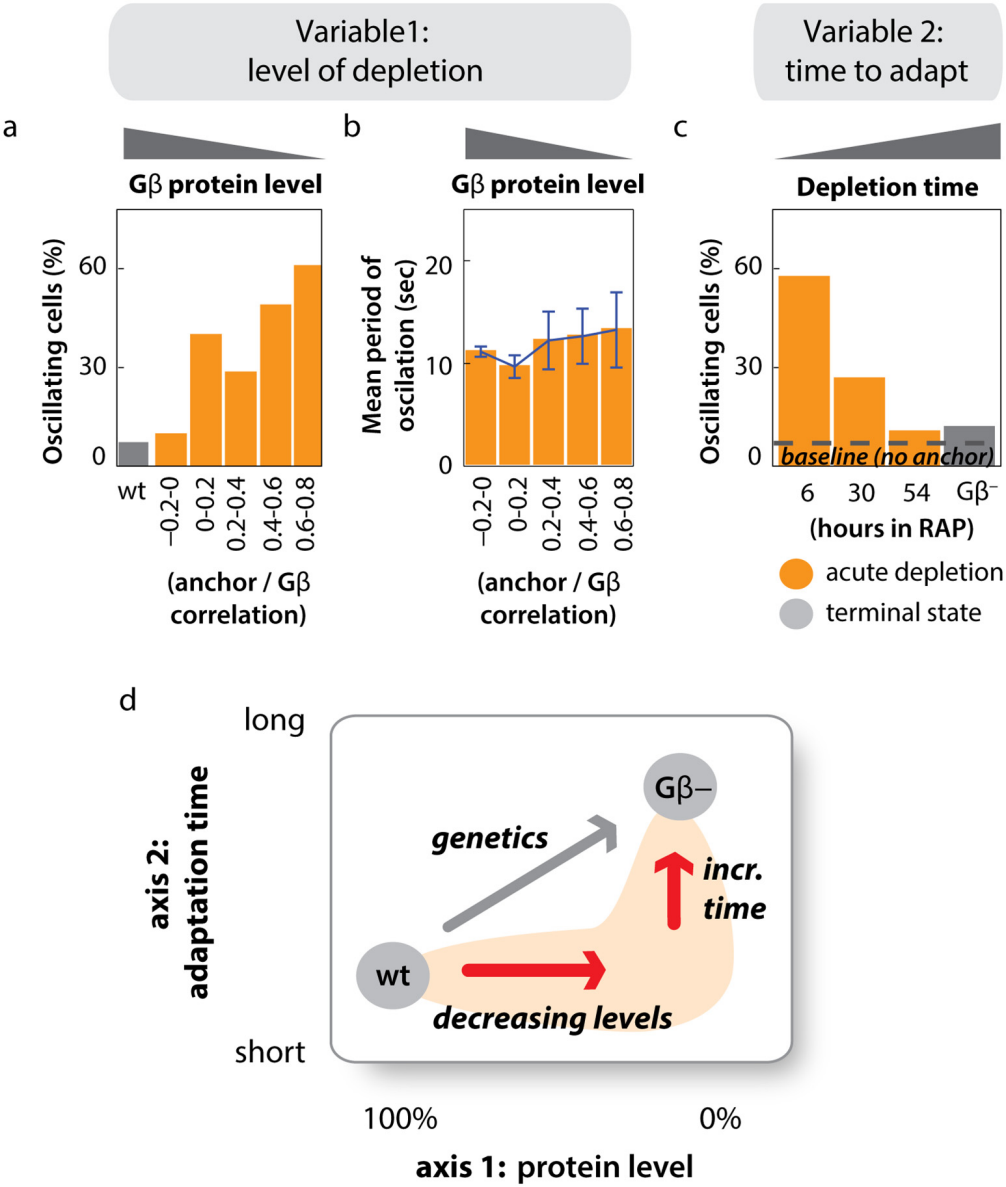
Actin oscillations depend on the extent and timing of Gβ sequestration. (A) Higher levels of sequestration (lower concentration of active Gβ) result in a larger fraction of oscillating cells. To achieve stable, intermediate levels of Gβ sequestration, cells were cotreated with 300 nM rapamycin and 0, 5, 10, 20 or 40 μM FK506, a competitive inhibitor of rapamycin. The correlation between Gβ and the anchor signal was extracted from single cells of all treatment conditions, and cells with similar levels of correlation were analyzed together (see S7 Fig). Gβ-unsequestered cells (wt) were analyzed for comparison. Negative correlation values indicate anticorrelation of Gβ and anchor in the unsequestered state. The mean of at least 20 cells in each bin is shown. Raw data can be found in S1 Data. (B) Higher levels of sequestration (a lower concentration of active Gβ) do not affect the period of oscillation. Cells and treatment conditions are the same as analyzed in (A). (*n* ≥ 20 cells per sequestration bin; plotted are means +/- stdev). The amplitude of actin oscillations is also not affected by sequestration of Gβ (S8 Fig). Raw data can be found in S1 Data. (C) The percentage of oscillating cells decreases over time during Gβ sequestration and approaches the terminal Gβ-null state. Cells were incubated with 1 μM rapamycin, and the fraction of oscillating cells was determined at the timepoints indicated (*n* > 25 Gβ-sequestered cells per condition). Raw data can be found in S1 Data. (D) Acute inhibition via rapamycin mediated protein sequestration can reveal phenotypes that are not accessible through classic genetic perturbations. First, it can reveal consequences of protein depletion to intermediate levels, such as the gradual or all-or-none emergence of phenotypes (axis 1). Second, rapid inactivation can reveal immediate phenotypes that are not accessible to slower methods of gene inactivation (axis 2).

Individual cells transition abruptly into the oscillatory mode. Oscillations become apparent as soon as rapamycin-induced sequestration of Gβ can be observed (S6 Fig and S3 Movie) and can continue for days (see later; Fig 4C and S1 Data). By treating cells with both rapamycin (the FKBP-FRB heterodimerizer) and a competitive inhibitor of heterodimerization (the small molecule FK506, an FKBP-FKBP homodimerizer), we titrated Gβ levels over the full dynamic range of the sequestration system (S7 Fig). As the amount of sequestered Gβ is increased, the properties of the oscillating state such as its period and amplitude did not change (S8 Fig). The oscillations have characteristics of an all-or-none behavior: only the percentage of oscillating cells increased (Fig 4A and 4B, S1 Data).

These phenotypes—whole-cell oscillations and traveling waves of actin polymerization—are reminiscent of previously observed actin-based activator—inhibitor systems [1,2,5,6,16–20]. However, the oscillations we observe here are triggered, persistent, and have an unusually large spatial range and high amplitude. This suggests that acute loss of Gβ pushes the cytoskeleton into an unusual state.

### Acute Inactivation of Gβ Differs from Gβ-Null Cells

Our observation that cortical F-actin oscillations follow acute sequestration of Gβ raises a key question: why did previous Gβ-null analyses fail to uncover this striking cytoskeletal phenotype? Consistent with published work [2,3], we find that very few Gβ-null cells display LimE-GFP oscillations cells (Fig 4C and S1 Data). We reasoned that if cells compensate for the loss of Gβ function over time, the phenotype induced by acute sequestration of Gβ should approach the Gβ-null phenotype after sufficient time has passed. Consistent with this hypothesis, the fraction of oscillating cells decreases over days of continuous Gβ sequestration and eventually approaches the small fraction seen in Gβ nulls (Fig 4C and S1 Data). Similar compensatory phenomena have been previously observed in other *Dictyostelium* signaling contexts. For example, the effect of LY294002, a PI3K inhibitor, on *Dictyostelium* cell migration fades during prolonged treatment [2], likely due to compensation by redundant signaling pathways [35]. In another case, the actin nucleator WASP relocalizes to the leading edge and compensates for SCAR/WAVE function when SCAR/WAVE is deleted [6]. Our findings suggest that a compensatory mechanism is also at work here: the globally oscillating state is suppressed in Gβ-null cells.

Our results highlight the value of using acute inhibition to uncover protein function. We have used rapamycin-induced Gβ sequestration to interrogate loss-of-function phenotypes along two “axes” (Fig 4D). By titrating the amount of sequestration while retaining its fast timescale (axis 1), it is possible to interrogate how a phenotype emerges, distinguishing between an all-or-none or gradual transition. Conversely, varying the timescale of perturbation (axis 2) reveals whether phenomena such as cellular compensation can mask an acutely induced phenotype. Applied to Gβ sequestration, we find that a new phenotype—a globally oscillating F-actin cytoskeleton—can be uncovered at points in this “phenotypic space” that are not accessible to standard genetic perturbations.

### Whole-Field Oscillations Emerge by Synchronizing Preexisting Oscillators

Multiple oscillating actin foci localize around the cell periphery on the basal surface of chemotactic cells. These foci often originate from previously aborted pseudopods that remain attached to the substrate. Internal and external signaling inputs are thought to entrain these foci, but how their dynamics are controlled for this to happen remains unknown (e.g., oscillation dynamics are unchanged in Ras, PI3K, and Gβ nulls) [2]. The large-scale cortical actin oscillations we observe here are similar in period to the previously described oscillating foci (13 +/- 3 s versus 9 +/- 2 s, respectively), suggesting that these two forms of cytoskeletal dynamics may be closely related. Thus, we tested whether our acute sequestration of Gβ would reveal signaling control over these oscillatory actin foci.

**Fig 5:**
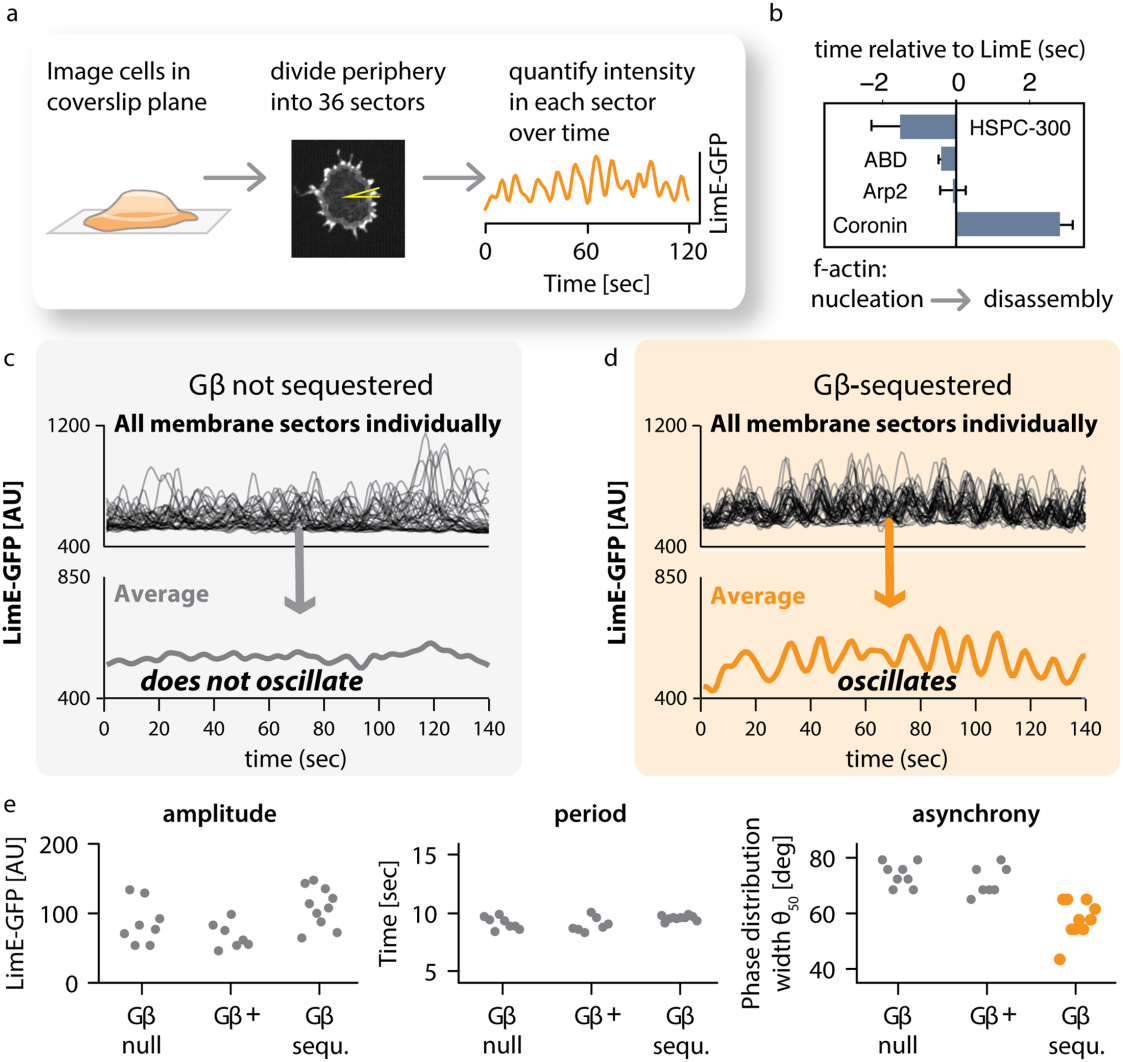
Gβ regulates coupling between individual actin oscillators. (A) Method of analysis. Cells expressing LimE-GFP were imaged by confocal microscopy in the plane where cells make contact with the coverslip. At each timepoint, the cell periphery was divided computationally into 36 sectors. For each sector, the intensity of LimE-GFP was quantified over time. The graph shows the trace for a single sector. (B) The temporal order of actin regulators at peripheral actin foci. The indicated actin reporters showed pulsatile behavior at the cell’s periphery. We measured their appearance, relative to LimE, in the same sectors in double-labeled cells. Oscillators from several cells (HSPC-300 *n* = 9; ABD *n* = 11; Arp2 *n* = 4; Coronin *n* = 4) were analyzed (plotted are means +/- SEM). An example for a dual color sequence of LimE and Coronin oscillations is shown in S10 Fig. Raw data can be found in S1 Data. (C) Traces for all 36 sectors of one control cell in which Gβ was not sequestered. Individual sectors oscillate, but the overall average does not. (D) Traces for all 36 sectors of one cell in which Gβ sequestration was induced by treatment with 1 μM rapamycin. Individual sectors oscillate, and so does the overall average. (E) Analysis of oscillation parameters. Each point represents the average of all 36 sectors of one cell. Amplitude and period of oscillations are similar in unsequestered (Gβ+), Gβ-sequestered (Gβ-sequ.), and Gβ-null cells. In contrast, synchrony of oscillations is increased in acutely sequestered cells. Raw data can be found in S1 Data. Further data are presented as supplements: a histogram of oscillation periods of individual sectors for one cell for each condition is shown in S9 Fig. To extract and compare phase information, we used the Hilbert transform, as shown in S11 Fig. A histogram of phase distributions for one cell each is shown in S12 Fig. Membership of individual oscillating sectors with the phase locked consensus is fluid as shown in S13 Fig.

To analyze individual actin foci, we collected confocal movies imaged in the plane where cells contact the coverslip. We developed a computational approach to comprehensively track and quantify the dynamics of actin foci by automatically identifying each cell’s periphery, subdividing it into ten degree sectors (thereby generating 36 tracked regions per cell), and measuring the mean intensity in each sector over time (Fig 5A). Consistent with previous results [2], we found large-amplitude oscillations in LimE-GFP intensity in some sectors (Fig 5A, right panel) but not others, with a mean period of approximately 10 s (S9 Fig). Regulators of F-actin formation localize to the same structures and oscillate as well: the peak of actin-nucleating SCAR/WAVE complex member HSPC-300 precedes that of LimE by about 2 s; Arp2 and the F-actin binding domain (ABD) of ABP120 peak at about the same time as LimE; and the peak of Coronin, a regulator of actin disassembly [2,62], lags behind LimE by more than 2 s (Fig 5B, S10 Fig, and S1 Data). These data suggest that focal LimE oscillations report cycles of polymerization and disassembly of F-actin.

We next addressed how the dynamics of actin foci compare between wild-type (Gβ-unsequestered) cells and Gβ-sequestered cells that exhibit whole-field oscillation. In both cases, individual sectors oscillate. However, the mean LimE intensity across all sectors in Gβ unsequestered cells does not show a marked oscillatory behavior (Fig 5C), whereas the mean intensity of sectors in Gβ-sequestered cells clearly oscillates (Fig 5D). Thus, the whole-field oscillations we observe upon Gβ-sequestration in the middle plane of cells (Fig 3A) are also reflected in the behavior of membrane-plane actin foci.

What properties of these individual oscillators change as cells transition to whole-field oscillation? We reasoned that changes in the amplitude, period, or the synchronization in phase between individual oscillating sectors could be responsible. We developed an automated approach using the Hilbert transform [63,64], which has been used extensively to analyze neuronal activity [65,66], to quantify the amplitude, period, and phase of individual oscillators over time (S11 Fig). Using this algorithm, we extracted the oscillation phase (i.e., whether currently at a peak or trough) as well as the instantaneous period (i.e., how fast the phase is changing) at each timepoint. Strikingly, only the phase synchrony differs in Gβ-sequestered cells (Fig 5E, S12 Fig and S1 Data). Yet although synchrony increases, it is not perfect: individual sectors can fall in and out of phase with the group over time (S13 Fig). Taken together, our data suggest that global oscillations in Gβ-sequestered cells are caused by increasing synchronization among preexisting membrane oscillators.

### Spatial Coupling Bypasses Established Cytoskeletal Signaling Pathways

**Fig 6:**
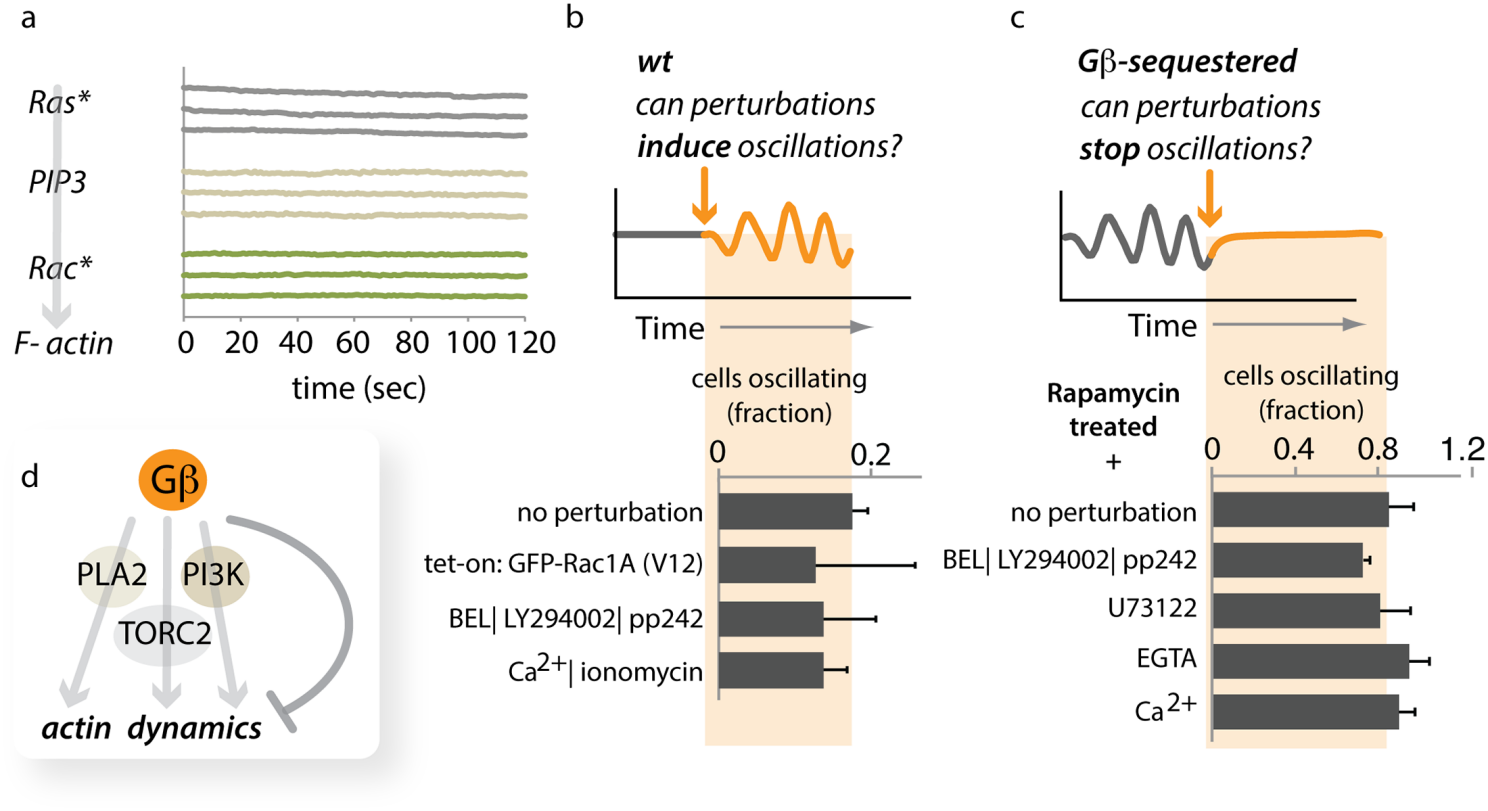
Gβ-mediated coupling bypasses established signaling pathways. (A) Signaling activities upstream of F-actin formation do not show global oscillations upon sequestration of Gβ. Representative traces (changes in intensity of reporter constructs in the cytoplasm) of several movies are shown for Ras activity (Ras*) visualized with YFP-RBD(PI3K1), PIP_3_ visualized with PhdA-GFP, and Rac activity (Rac*) visualized with GBD(PAK)-YFP. (B) Perturbations of core chemotactic regulatory pathways do not induce global oscillations of LimE. Neither induced expression of a constitutively active version of Rac1A (GFP-Rac1A-V12) nor a triple drug cocktail (BEL|LY294002|pp242) inhibiting PLA2-, PI3K-, and TORC2-mediated signaling induces global oscillations of LimE-RFP in wild-type (Ax2) cells. Similarly, increasing the intracellular concentration of Ca^2+^ has no effect. Data represent the means of more than 35 cells from at least 2 d for each condition (+/- stdev). Raw data can be found in S1 Data. (C) Inhibition of core chemotactic regulatory pathways does not abolish Gβ mediated global oscillations of LimE-GFP. Gβ-sequestered, oscillating cells were treated with various drugs to determine their effect on oscillatory behavior. Neither a triple-drug cocktail (BEL|LY294002|pp242) that simultaneously blocks PLA2-, PI3K-, and TORC2-mediated signaling, nor unbalancing Ca^2+^ levels (blocking PLC with U73122, supplying Ca^2+^ or chelating any Ca^2+^ present in the buffer with EGTA) had a significant effect on the presence of global LimE-GFP oscillations. Data represent the means of more than 25 cells from at least 2 d for each condition (+/- stdev). Raw data can be found in S1 Data. (D) Gβ appears to bypass established signaling pathways to regulate the spatial range of coupling. S19 Fig shows data to validate the use of pp242 as an inhibitor for TORC2 mediated signaling in *Dictyostelium* cells.

Downstream of Gβ, three signaling pathways, defined by PI3K, TORC2, and PLA2, are known to instruct actin-based motility in *Dictyostelium* (Fig 6D). Ras activity can feed into both PI3K and TORC2, and downstream, Rac activation is thought to connect these signaling modules to the actin cytoskeleton [31,33,38,56]. Enhanced activity of these pathways leads to wider, more stable zones of actin polymerization compared to the isolated oscillating foci.

We investigated whether Gβ uses any of these signaling pathways to regulate spatial coupling of actin foci. First, we analyzed the dynamics of Ras activity, PIP_3_ levels, and Rac activity in single cells. Gβ sequestration neither induced oscillations nor caused any other apparent changes to these signaling currencies on a timescale of minutes (Fig 6A). Second, we perturbed the activities of members of these pathways in wild-type cells to determine whether global LimE oscillations would emerge. Neither inducing Rac activity (Tet-On: GFP-Rac1A[V12]), blocking all three pathways (using a pharmacological cocktail: BEL|LY294002|pp242), nor raising the levels of intracellular Ca^2+^ (a messenger commonly oscillating in other systems [22,67]) led to global oscillations of F-actin (Fig 6B and S1 Data). Third, we interfered with these pathways in Gβ-sequestered oscillatory cells to determine whether their activity was required for synchrony. Acute inhibition of all three pathways caused only a very small decrease in the number of oscillating cells, while unbalancing Ca^2+^ levels did not inhibit global oscillations at all (Fig 6C and S1 Data). We conclude that Gβ’s control over the coupling range of actin oscillators likely involves a different, currently unidentified mediator.

### Increased Spatial Coupling of Oscillators Impairs the Establishment of Cell Polarity

How can hypercoupling between cytoskeletal oscillators lead to a defect in directed cell migration? The coupling state among the oscillators might be an important parameter for upstream cues to polarize the cytoskeleton—a prerequisite for cell motility. To investigate this question, we tracked individual Gβ-sequestration cells over time, simultaneously monitoring cytosolic actin dynamics and cell migration in both the presence and absence of rapamycin.

**Fig 7:**
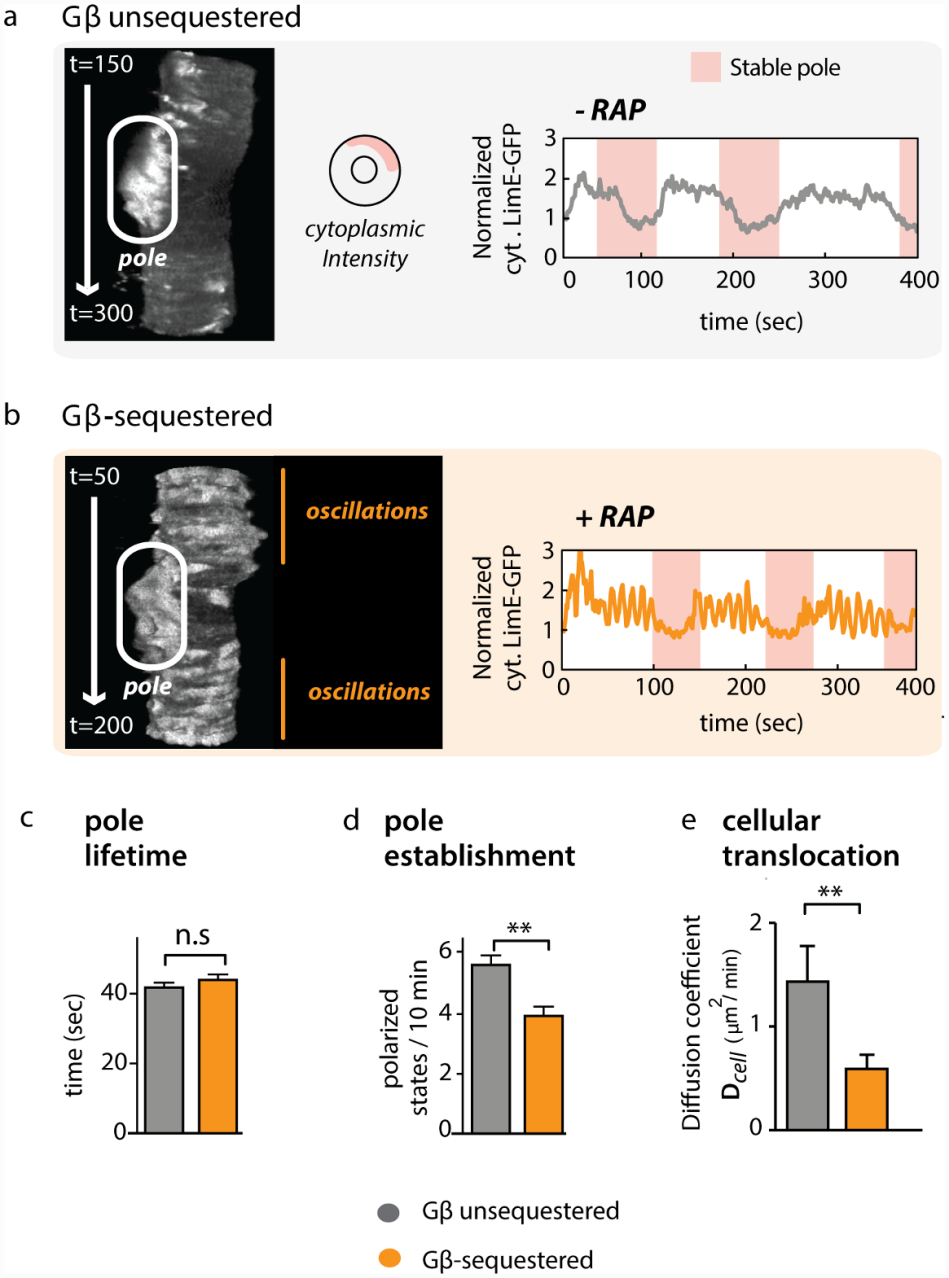
A hypercoupled cytoskeleton competes with establishment of cell polarity. (A) In Gβ unsequestered (wt) cells, phases of polarization, characterized by low cytoplasmic LimE-GFP intensity, alternate with apolar phases. A confocal slice from the middle of an unsequestered cell is stacked into a kymograph (t-stack), where the *y*-axis represents time and the *x*-axis represents intensity along the cell’s lateral surface. Continuous bright areas (white oval) indicate LimE-GFP accumulation in a pseudopod. The corresponding trace of cytoplasmic LimE-GFP intensity on the right shows that phases of polarity (pink shading; see S14 Fig) coincide with low levels of cytoplasmic reporter (and, therefore, higher levels of polymerized actin at the periphery). A total of 28 unsequestered cells were analyzed with similar results. (B) Whole-field LimE-GFP oscillations are restricted to apolar phases in Gβ-sequestered cells. One representative t-stack and corresponding trace is shown. A total of 46 Gβ-sequestered cells were analyzed with similar results. S15 Fig shows that the strength of polarization is similar between Gβ-sequestered and Gβ-unsequestered cells. (C) The lifetime of poles in Gβ-unsequestered cells (grey; *n* = 28) and Gβ-sequestered cells (orange; *n* = 46) is similar (*p* = 0.51 for their difference, Student’s two-tailed *t* test; plotted are means +/- SEM). Raw data can be found in S1 Data. (D) The frequency at which poles are established in Gβ-sequestered cells is reduced compared to Gβ-unsequestered cells (the same set of cells as in Fig 4C is analyzed; plotted are means +/- SEM; *p* < 10^-4^, Student’s two-tailed *t* test). Raw data can be found in S1 Data. (E) Cellular translocation is slowed down for Gβ-sequestered cells (mean +/- SEM = 0.59 +/- 0.14 μm^2^/min, *n* = 46) compared to Gβ-unsequestered cells (mean +/- SEM = 1.43 +/- 0.35 μm^2^/min, *n* = 28; *p* < 0.003, Student’s two-tailed *t* test). Mean squared displacement is a suitable metric for cell migration over short periods of time (S20 Fig). Raw data can be found in S1 Data. S18 Fig shows that oscillating actin foci are suppressed during cell polarization in wild-type cells. Abbreviations used: SEM = standard error of the mean; n.s. = not significant, *p*-value > 0.05; ** indicates a highly significant *p*-value of < 0.01.

For this analysis, we returned to confocal imaging in the midplane of the cell. Here, polarization events are distinguished by a relatively stable actin patch that coincides with a substantial drop in cytoplasmic LimE-GFP reporter levels (Fig7A and 7B and S14 Fig). In both control and Gβ-sequestered cells, polarized patches are of similar intensity (S15 Fig), and phases of polarity alternate with apolar phases, which can easily be visualized in t-stack kymographs (Fig 7A and 7B; left panels). In this representation, the *y*-axis represents time, and the lateral surface of the cell is shown for each timepoint along the *x*-axis.

We found that Gβ-sequestered as well as Gβ unsequestered cells were capable of cycling between polarized and apolar states (S4 and S5 Movies). Consistent with our prior results, acute sequestration of Gβ induced large-amplitude oscillations of F-actin. However, long-term imaging revealed that these oscillations are largely restricted to apolar phases—times when the cell is not undergoing protrusion or migration (Fig 7B and S5 Movie). Thus, phases of polarization appear to be incompatible with whole cell oscillations. While increased coupling in Gβ-sequestered cells did not affect the lifetime of poles once they successfully formed (Fig 7C and S1 Data), Gβ sequestration significantly (*p* < 10^-4^, Student’s two-tailed *t* test) impaired the establishment of new poles (Fig 7D and S1 Data). Consistent with a reduced number of cell polarization events, sequestered cells translocate at a significantly reduced speed (*p* < 0.003, Student’s two-tailed *t* test, Fig 7E and S1 Data).

**Fig 8:**
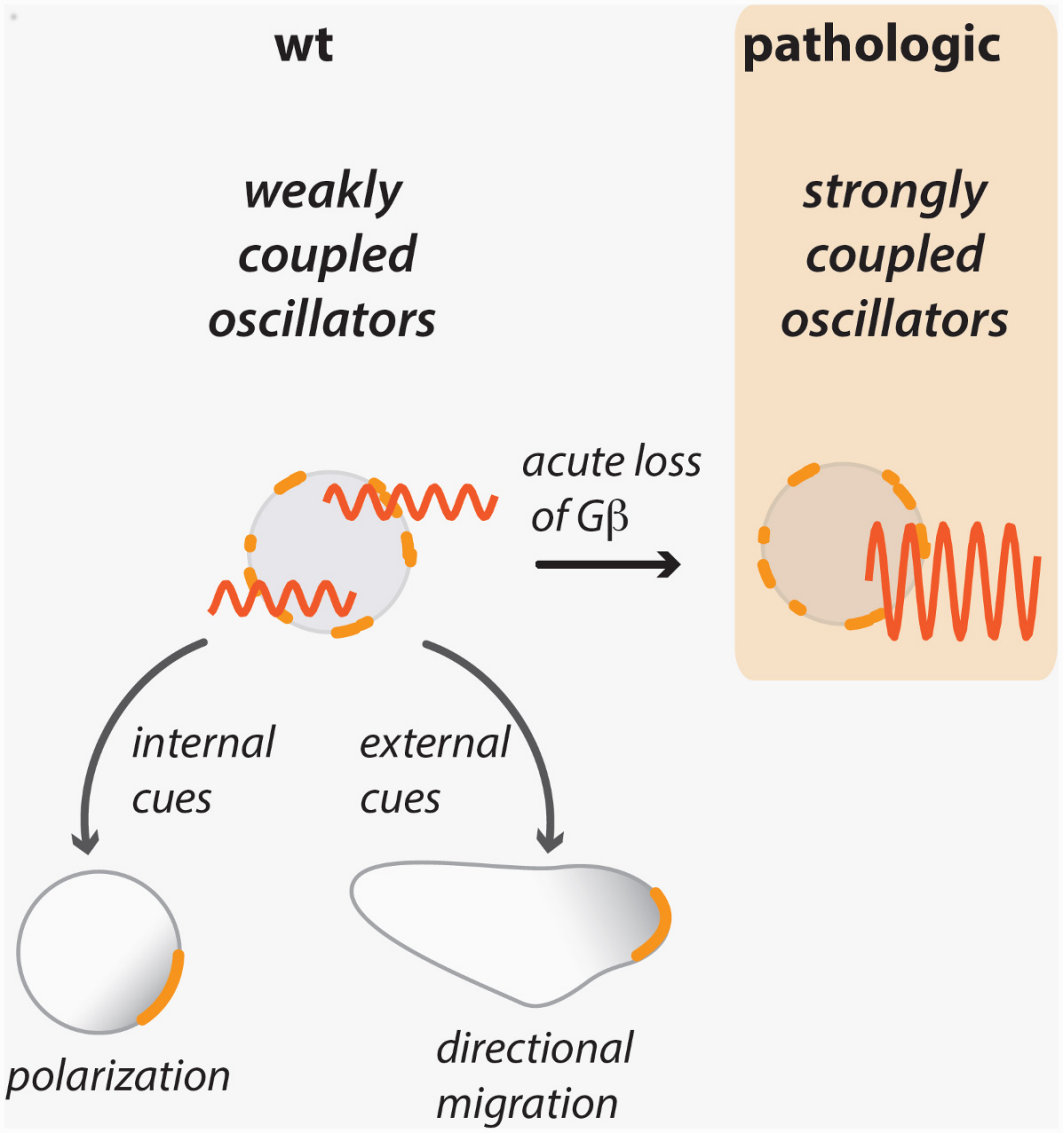
Acute loss of Gβ induces a hypercoupled cytoskeleton. By synchronizing weakly coupled peripheral oscillators, a hypercoupled state is induced that is apparent as whole-field oscillations. This pathologic state is less permissive to the establishment of cell polarity and continuous realignment of polarity in a gradient. We suggest that this hypercoupled state prevents oscillators from becoming patterned by upstream signaling cues from inside or outside the cell.

Taken together, our data show that appropriate control of coupling between localized cytoskeletal oscillators is essential for efficient polarization and motility as well as directional sensing. Increasing the strength of coupling—through acute loss of Gβ—synchronizes actin dynamics, which hampers the entrainment of the actin cytoskeleton by both internal polarity cues as well as entrainment by the external cues that are necessary to direct motility (Fig 8).

### Oscillator Coupling Is Sufficient to Increase Sensitivity to Noisy Inputs

One of the most remarkable features of chemotaxis is the ability of migrating cells to accurately sense extraordinarily shallow chemical gradients [68]. Previous work has suggested that the signaling network downstream of Gβ plays a crucial role in this input sensing [5,29,69,70]. Here, we have uncovered a separate link between Gβ and the cytoskeleton in tuning coupling between actin oscillators. Might oscillator coupling also play a role in input sensitivity?

**Fig 9:**
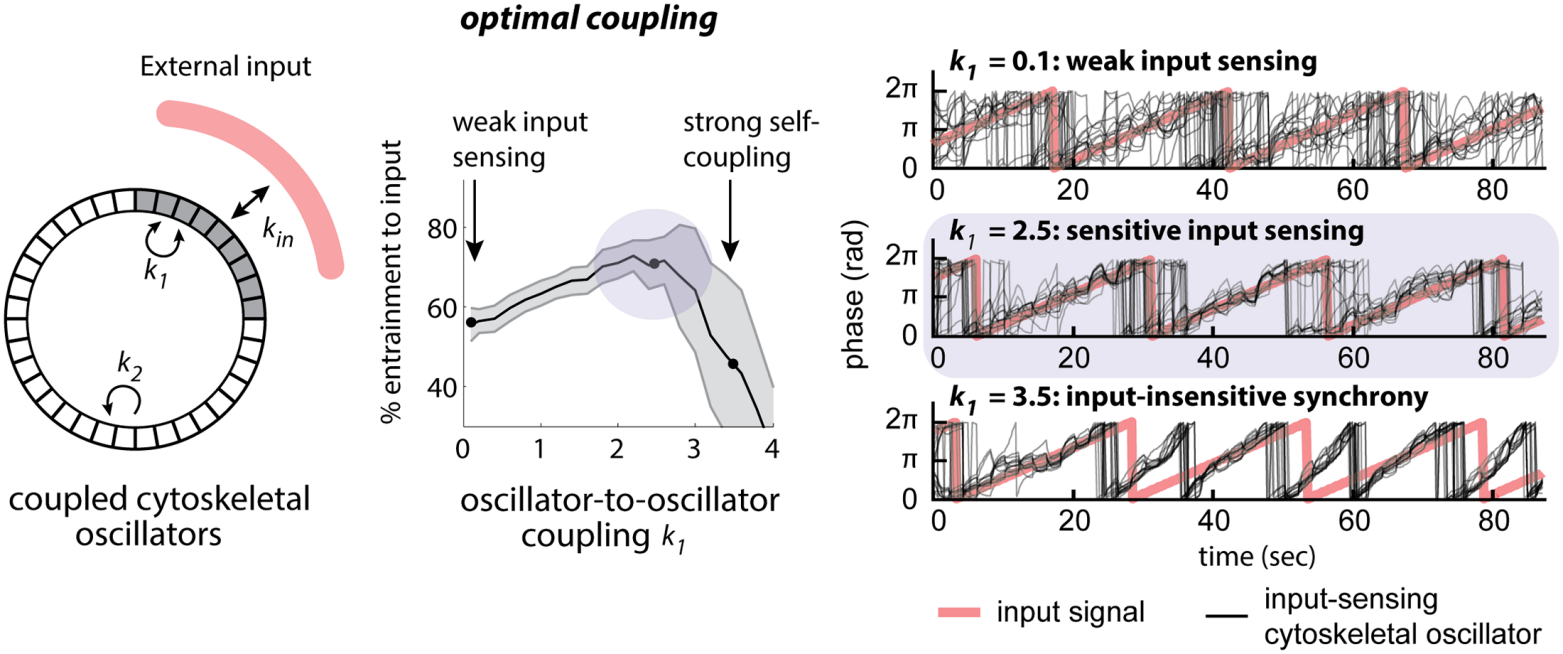
A mathematical model demonstrates that intermediate oscillator coupling is sufficient to increase sensitivity to noisy inputs. Wild-type cells may be in a range of optimal coupling between cytoskeletal oscillators to facilitate entrainment by signaling cues. To investigate how coupling strength influences signal detection, we have built a simple model in which sectors all around a circle couple at a strength *k*_*2*_. The test area couples locally with a strength *k*_1_, and entrainment to an external input of strength *k*_*IN*_ (left panel) is assessed. At intermediate values for oscillator coupling, *k*_1_ entrainment to the input is optimal (middle panel). Examples for oscillator entrainment at different values of *k*_1_ are shown (right panel).

We reasoned that oscillator-to-oscillator coupling might represent a means of sharing information between nearby regions of the cell periphery. By comparing noisy receptor—ligand interactions at multiple locations, cells might improve their ability to discriminate signal from noise when choosing a migration direction. To test this hypothesis in a simple context, we built a mathematical model representing input sensing at the cell’s periphery (Fig 9). It should be emphasized that this model is not meant to capture the full complexity of the cell’s gradient sensing and chemotaxis pathways, but rather represents a minimal model to quantitatively interrogate the essential elements of oscillator-to-oscillator coupling and entrainment to an input. Our model incorporates a circular lattice of actin oscillators representing the cell’s cortex. Oscillators are coupled to one another by a term that increases sinusoidally with their difference in phase [71] and can also be coupled to an oscillating input signal using the same mechanism. Although the chemoattractant signals presented to a real cell are unlikely to oscillate in this fashion, the exact mechanism for input coupling is unknown, and our simplifying assumption allowed us to model oscillator-to-input and oscillator-to-oscillator coupling in a single unified framework. Our model includes three parameters that define the coupling between an external input and the nearby membrane (*k*_*IN*_) and the coupling between membrane oscillators (parameters *k*_*1*_ and *k*_*2*_ for input-coupled and non-input-coupled membrane oscillators). We also include a term (σ) to represent noise in input-to-oscillator coupling.

Our model reproduced well-known features of coupled oscillator systems. Increasing oscillator-to-oscillator coupling showed an abrupt transition to global synchrony, consistent with prior work modeling the synchronization of weakly coupled oscillators as a phase transition (S16 Fig) [68,69]. This is analogous to the effect observed after Gβ sequestration, in which the transition to global oscillation appears to be all-or-none in individual cells (Fig 4 and S1 Data).

To test how coupled oscillators are affected by features of the input signal, we set out to determine how oscillator-to-oscillator coupling affected sensing of weak inputs (low values of *k*_*IN*_) or noisy inputs (high values of σ). We found that increasing coupling could not improve sensing of weak noise-free inputs but rather led to spontaneous synchronization as coupling strength is increased (S21 Fig). In contrast, oscillator-to-oscillator coupling markedly improved sensing of noisy inputs (Fig 9). For simulations with little or no coupling, the effect of noise was dominant, and membrane oscillators were unable to accurately couple to inputs (Fig 9; *k*_*1*_ = 0.1). Conversely, for very strong coupling, oscillators became synchronized to one another so strongly that they were completely input-insensitive (Fig 9; *k*_*1*_ = 3.5) [25,71]. Between these two extremes, our model revealed an optimum of input sensitivity at an intermediate coupling strength (Fig 9; *k*_*1*_ = 2.5).

If weak oscillator-to-oscillator coupling was indeed beneficial for input sensing, one would expect wild-type cells to exhibit some coupling between oscillating foci. Indeed, we find experimentally that in wild-type cells the relative phases of oscillators are not random but loosely correlated (Fig 5E, asynchrony; phase distribution width Θ_50_ < 90 [deg.]; S12 Fig and S1 Data). Thus, we propose that upstream signaling cues optimally entrain the cytoskeleton when the coupling strength between its dynamic units is of intermediate strength.

## Discussion

### Spatial Coupling between Cytoskeletal Units Regulates Polarity and Directional Migration

A dynamic actin cytoskeleton drives eukaryotic cell migration. Waves, flashes, patches, and oscillatory actin foci have been observed in *Dictyostelium* [2,5,6,15–19], neutrophils [1,4], and other mammalian cells [17,20,72,73]. Underlying these phenomena are nonlinear reaction processes that exhibit a range of behaviors including excitability and oscillations [1,2,26,29,70,74]. These cytoskeletal dynamics are shaped further by upstream cues such as internal polarity signals [1–3] and external chemoattractant [4,5]. Here, we show that signaling also directly regulates the strength of coupling between local cytoskeletal processes. Acute loss of Gβ leads to strong synchronization of actin oscillators, which has detrimental consequences for cell polarity, motility, and directionality.

Electrotaxis revealed this new role for Gβ in directed cell migration. However, we expect the link between Gβ and cytoskeletal dynamics to be essential for interpreting other cues as well. During chemotaxis, when the activity of the Gαβγ heterotrimer is proportional to the amount of the chemical signal the cell experiences [75], fine control of the magnitude and intracellular distribution [76] of oscillator coupling may be possible.

In future work, it will be important to learn more about how Gβ exerts this control. Common signaling pathways involving PI3K, TORC2, and PLA2 appear to be not essential. Similarly, perturbing levels of Ca^2+^, a messenger known to oscillate in many systems [22,67], including chemotaxing *Physarum polycephalum* cells [77], shows no obvious effect on coupling between actin oscillators. *P*. *polycephalum*, however, is a beautiful, conceptual precedent for the idea that cell movement may be governed by the coupling between independent oscillators: in this organism, periodic streams of small pieces of cytoplasm can become entrained to each other, which, through further modulation by attractants or repellants, supports directional movement [78].

Recent evidence in *Dictyostelium* shows that Gβ interacts with Elmo, which suggests a possible direct link to the cytoskeleton bypassing the other signaling pathways [79]. We observed oscillation of HSPC-300, a member of the actin-nucleating SCAR/WAVE complex. This may be the most upstream oscillator, with F-actin reporters and disassembly factors (e.g., Coronin) following its dynamics. In this case, SCAR/WAVE’s relevant regulators will need to be identified [80]. Mechanistically, how could loss of Gβ increase the strength of coupling? Based on the mechanisms through which oscillators are coupled in other systems, possible explanations include (1) increasing the density of oscillators at the periphery while keeping the coupling range of each constant [81], and/or (2) directly increasing the range of a diffusible or mechanical signal that is generated by the oscillators [26]. Our experimental data support the first hypothesis. In strongly coupled Gβ-sequestered cells, a larger fraction of sectors contain actin oscillators compared to Gβ-unsequestered cells or Gβ-null cells (S17A Fig). Moreover, upon Gβ sequestration, the number of membrane sectors that contain an actin oscillator increases, while the amplitude of the oscillators remains constant (S17B Fig). Additionally, we find that during cell polarization, oscillators largely disappear from the sides and back of the cell (S18 Fig). Taken together, our data suggest that the number or density of oscillators is regulated, and this may be used as a mechanism to control coupling strength. Additional mechanisms could affect the firing threshold or the refractory period of the oscillators.

Our simple mathematical model helps to guide intuition on why coupling between oscillators could be advantageous for polarity and directional movement. For both cases, the signals that need to be interpreted can be noisy, and in these scenarios moderate coupling between oscillators can provide an advantage—input-coupled oscillators can “share” information to filter noise and better entrain to an input signal. Our results are consistent with recent predictions in bacterial chemotaxis, in which an optimal membrane distribution of receptors balances sensitivity to spatially correlated external noise and spatially uncorrelated intrinsic noise (which can be filtered out by a similar mechanism of local information sharing) [82].

### The Benefits of Acute Perturbations: A Novel Cytoskeletal Role for Gβ

Our work highlights limitations in classical genetic approaches. Genetic nulls are the most common means of assaying gene function in *Dictyostelium*. However, many genetic mutants give no or mild phenotypes, and they often require combined hits in multiple signaling pathways to significantly inhibit chemotaxis [6,32–36]. In theory, two mechanisms can account for this: a selection on the population levels can favor a subset of cells (potentially carrying suppressor mutations) that best cope with the genetic change. Alternatively, intrinsic redundancy with parallel pathways or slow compensation via negative feedback can obscure the true role of a gene in cell behavior [42]. Such compensation enables robust function and is a widely employed characteristic of adaptive/homeostatic systems. For example, pharmacological inhibition of synapses transiently inhibits signal transmission, but homeostatic mechanisms restore function within minutes [39–41]. The motor of bacteria is another example. It compensates for persistent changes in the level of internal signaling components to maintain the robustness of chemotaxis [41].

Which mechanism is at play in our case? Both Gβ-null knockout and wild-type cells lack excessive coupling of actin oscillators, albeit likely for different reasons. In wild-type cells, Gβ suppresses the coupling, while Gβ-null knockout cells have, over time, arrived at a Gβ independent steady state that does not support oscillations. Transformation of Gβ-null knockout cells with the sequesterable Gβ construct restores wild-type physiology, which can become transiently unbalanced upon acute Gβ sequestration. This imbalance remains for days—long enough for us to observe its effect on oscillator coupling—but eventually the steady state of Gβ-null knockout cells is assumed again, potentially due to compensation from parallel pathways. We favor this possibility over genetic suppression based on the speed with which oscillations disappear again after induction.

As a consequence of compensation, different modes of gene inactivation can result in strikingly different phenotypes. In zebrafish, gene knockdowns can produce strong phenotypes that are masked by compensation in genetic knockouts [83]. Our data suggest that additional phenotypes appear when proteins become inactivated even more rapidly. Gβ-knockout and knockdown cells have been extensively studied in *Dictyostelium* and other systems [43,44,84]. Although defects have been reported for a wide range of chemoattractant-stimulated responses, including directed migration [44], these cells display normal basal polarity and actin dynamics [2,3]. Acute sequestration was essential to uncover the role of Gβ in tuning cytoskeletal dynamics and initiating cell polarity. In this light, our work suggests that much can be learned by revisiting classical mutants with acute perturbation approaches, and not only in instances in which a loss-of-function mutation is lethal.

## Materials and Methods

### Dictyostelium Cell Culture and Sequestration Experiments

*Dictyostelium* strains were grown at 22°C in HL5 medium (ForMedium) in Nunclon tissue culture dishes or in suspension in flasks shaken at 180 rpm. Cells were routinely used from nonaxenic cultures. In this case, cells were grown in association with *Klebsiella aerogenes* (K.a.) on SM agar plates and used for assays when bacteria began to get cleared [85]. Growth under these conditions gave the strongest responses to stimulation with folate, so this condition was used for most subsequent rapamycin-mediated sequestration experiments. However, sequestration of Gβ also induced oscillations in F-actin when cells were grown in HL-5 instead. For imaging experiments, a scrap of cells was seeded in 200 μl HL5 in a Lab-Tek II 8 well chamber (Nunc), allowed to settle, and washed one to two times in KK2 (16.5 mM KH_2_PO_4_, 3.9 mM K_2_HPO_4_, 2 mM MgSO_4_) immediately before the assay. Rapamycin (SIGMA) was freshly prepared at 2 μM in KK2 and added 1:1 in sequestration experiments. To render cells responsive to cAMP (Fig 1C), aggregation-competent amoebae were prepared by resuspending washed cells at 2 x 10^7^ cells/ml in KK2, starving them for 1 h while shaking at 180 rpm, followed by pulsing the cells with 70–90 nM cAMP (final conc.) for another 4 h. Before stimulation with 1 μM cAMP, cells were basalated (shaking at 180 rpm in the presence of 5 mM caffeine for 20 min) with or without 5 μM rapamycin, washed in ice-cold KK2, and kept on ice until stimulation. For analysis by western blotting, samples were resolved on 4%–15% SDS-PAGE gels. After electrophoresis, proteins were transferred to PVDF membrane and probed with anti—phospho-PKC (pan) antibody from Cell Signaling Technology (190D10), which was used to detect the activation loop (T309) phosphorylation of PKBR1, and anti—pan-Ras antibody from EMD (Ab-3). Sequestration of Gβ was confirmed by fluorescence microscopy.

### Constructs and Strains

PhdA-GFP, LimEΔcoil-GFP, LimEΔcoil-RFP, GFP-Arp2, Hspc300-GFP, ABD-GFP, GBD(PAK)-YFP, Coronin-GFP, and YFP-RBD(PI3K1) have been described previously [2,5,35,86]. Standard methods of molecular biology, including reagents from Quiagen and Zyppy Plasmid Miniprep Kits from Zymo Research, were used to generate the following constructs: SRC-YFP-FRB (pHO34) was assembled in pDXA-YFP by subcloning FRB (XhoI/XbaI) from pOW578 with a synthetic sequence (HindIII/Nsi1) encoding the myristoylation tag from SRC. cAR1-RFP-FRB (pHO39) was assembled in pDXA-YFP by replacing YFP with a fragment containing cAR1-RFP (HindIII/XhoI) and adding amplified FRB (XhoI/XbaI). CalnexinA-CFP-FKBP (pHO232) was assembled in multiple steps. CalnexinA was amplified from a published plasmid [55] and inserted into a variant of pDXA-YFP encoding FKBP (pHO167) or CFP and FKBP (pHO232). A Gateway-compatible vector derived from pDM448 [87] encoding FRB-RFP was generated (pHO436), into which Gβ was inserted with an LR reaction to build FRB-RFP-Gβ (pHO536). A tetracycline-inducible variant of GFP-Rac1AV12 (pHO578) was built by enzymatic assembly (Gibson) in pDM369 [87].

To generate stable cell lines, cells were transformed by electroporation (Genepulser Xcell, Bio-Rad) using 10–20 μg DNA per 4x10^6^ cells (100 μl) in 1 mm cuvettes (Bio-Rad). Two consecutive pulses with a 5-s recovery period between were delivered at 750 V, 25 μF, and 50 Ohm. For overexpression, cells were plated in bulk and selected with G418 (10 μg/ml) and/or hygromycin (50 μg/ml) the next day.

The time course of inducible sequestration (Fig 1B) was benchmarked in strain HO543: A Gβ-null strain (LW6) derived from DH1 [44] was used as the base strain into which the sequestration system was engineered. First, pHO536 (FRB-RFP-Gβ) was introduced, and transformants were selected with hygromycin (50 μg/ml) to give *HO535*. This strain was then transformed with pHO167 (calnexinA-YFP-FKBP) to give *HO543* or simultaneously with pHO232 (calnexinA-CFP-FKBP) and LimEΔcoil-GFP to give *HO547*, with pHO232 and phdA-GFP to give *HO548*, with pHO232 and pOH250 to give *HO549*, and with pHO232 and PAK(GBD)-YFP to give *HO630*. Additional anchors, such as a NLS or the transmembrane domain of Miro, were tested, but yielded poor depletion of Gβ. Transformants were selected with G418 (10 μg/ml). When appropriate for comparison, parent strains DH1 expressing LimEΔcoil-GFP (*HO618* **)** or LW6 expressing LimEΔcoil-RFP (*HO595*) were analyzed. The following strains were used to control for the effect of rapamycin mediated recruitment: DH1 expressing LimEΔcoil-GFP, pHO232 and pHO39 (*HO620*; G418 resistant), Ax2 (Kay lab) expressing LimEΔcoil-RFP, pHO232 and pHO34 (*HO621*; G418 resistant) and Ax2 (Kay lab) expressing LimEΔcoil-GFP, pHO232 and pHO536 (*HO626*; G418 and hygromycin resistant). For dual color oscillation experiments (Fig 5B), Ax2 (Kay lab) cells expressing LimEΔcoil-RFP together with GFP-Arp2 (*HO632*), Hspc300-GFP (*HO634*), ABD-GFP (HO638), or Coronin-GFP were analyzed.

### Microscopy

A spinning disc Nikon Eclipse Ti fitted with a spinning disc head, 405 nm, 488 nm, and a 561 nm laser line and appropriate emission filters were used to record CFP, RFP, and GFP (or YFP) double- or triple-labeled cells at room temperature. Images were routinely recorded using a 60x (1.45 NA) objective, a Clara Interline CCD camera (Andor Technologies), and NIS Elements software. After analysis, when necessary for presentation, contrast was adjusted uniformly using ImageJ or Photoshop, and to image sets of some experiments a uniform Gaussian Blur was applied. To quantify oscillations, a single two- or three-channel image was taken to assess Gβ sequestration, followed by a 2-min movie (1 frame/second) to record behavior in the reporter channel at the lowest laser intensity necessary for reasonable signal-to-noise. Longer imaging periods (10 min) and/or adjustment of the focal plane close to the coverslip were used when necessary (e.g., to record individual oscillating foci or alternating polar and apolar states).

### Drug Treatments

For Fig 5, Ax2 cells expressing LimE-RFP were analyzed for 2 min (1 frame/second) immediately before and for 2 min (within 5 min) after applying perturbations. For Gβ sequestration, only oscillating cells (strain *HO547*) were considered. Ca^2+^ and ionomycin were used at 10 mM and 10 μM, respectively. For triple drug inhibition, Bromoenol lactone (BEL 5 μM) was washed out after 5 min of treatment, after which acute application of LY294002 (50 μM) together with pp242 (40 μM) followed. BEL and LY294002 have been demonstrated as effective inhibitors of PLA2 and PI3K in *Dictyostelium* before [38]; pp242 is an inhibitor of TOR kinase and inhibits TORC2-mediated phosphorylation events in *Dictyostelium* (S19 Fig). Expression of tet-on GFP-Rac1A(V12), was induced overnight with 100 μg/ml doxycycline. The effect on oscillating, Gβ-sequestered cells was additionally tested by treatment with U73122 (5 μM), EGTA (10 mM), and Ca^2+^ (10 mM).

### Electrotaxis Experiments

The electric fields were applied as previously described for vegetative *Dictyostelium* cells [88] by using μ-Slides (Ibidi). These tissue-culture-treated slides with small cross-sectional area provide high resistance to current flow and minimized Joule heating during experiments. To eliminate toxic products from the electrodes that might be harmful to cells, agar salt bridges made with 1% agar gel in Steinberg’s salt solution were used to connect silver/silver chloride electrodes in beakers of Steinberg’s salt solution to pools of excess developing buffer (5 mM Na_2_HPO_4_, 5 mM KH_2_PO_4_, 1 mM CaCl_2_, and 2 mM MgCl_2_, pH 6.5) [89] at either side of the chamber slide. EF strength is empirically chosen (~10V/cm) based on our previous experience [90] and measured by a voltmeter before and after each experiment. Fields of *HO547* cells were chosen based on the presence of Gβ and anchor expressing cells, which were distinguished by fluorescence imaging (see Microscopy section for details). High-definition DIC movies (1 frame/30 s) were recorded at room temperature for at least 30 min after the electric field was switched on. To quantify directionality and speed, time-lapse images were imported into ImageJ (http://rsbweb.nih.gov/ij). Tracks were marked by using the MtrackJ tool and plotted by using the Chemotaxis tool described [91]. All experiments were repeated and produced similar results. Data are combined and presented as means +/- SEM (standard error). To compare group differences, unpaired, two-tailed Student’s *t* test was used. A *p*-value of less than 0.05 is considered significant.

### Folic Acid Chemotaxis Experiments

HO543, DH1, or LW6 (Gβ null) cells were grown in HL5 medium containing 20 μg/ml G418 and 50 μg/ml hygromycin. Two days before the experiment, 2x10^5^ cells were mixed with an overnight culture of K.a. in 250 μl streptomycin-free HL-5 medium and plated on an SM agar plate. On the day of the experiment, cells were washed off the SM plate with DB buffer, washed once, and resuspended in DB at 2x10^7^ cells/ml. Suitable amount of cells were transferred to LabTek II chambered coverglass (Nalge Nunc) containing DB with 5 μM rapamycin and 0.05% DMSO. For folic acid chemotaxis, Femtotips microcapillary pipettes (Eppendorf) filled with 1 mM folic acid were used. Microscopy for this set of experiments was carried out with a Nikon Eclipse TiE microscope illuminated by an Ar laser (YFP) and a diode laser (RFP). Time-lapse images in bright field, YFP, and RFP channels were acquired by a Photometrics Evolve EMCCD camera controlled by Nikon NIS-Elements. Tracks of cell migration were analyzed in ImageJ to obtain directedness and speed of cells.

### Automated Identification of Cells and Subcellular Regions

For all other analyses, cells were identified, tracked, and processed to extract various properties (e.g., cytoplasmic fluorescence, membrane fluorescence, extent of polarization, angle of polarization) using custom code written in Matlab. First, initial locations for each cell were provided by hand-drawn masks such that each mask contains a single cell at the first timepoint. At each subsequent timepoint, each cell was tracked by extracting a 100x100 pixel box centered at that cell’s prior location in the LimE-GFP fluorescent channel. To identify the cell within this box region, interior pixels were separated from background intensity using a fixed intensity threshold, followed by binary erosion with a single-pixel structuring element (to remove isolated noncell pixels) and a hole-filling operation (to fill all pixels within the cell). The largest connected component within this image was assumed to be the cell.

For each cell and at each timepoint, we extracted the following features:

- Centroid: The “middle” of the cell
- Center of mass: The intensity-weighted center of mass of the LimE-GFP channel (e.g., cells with a bright actin pole would have a center of mass biased toward the pole).
- Cytoplasmic intensity: The mean LimE-GFP intensity was extracted from a disk with a radius of 10 pixels, centered at the cell’s centroid.
- Cell membrane: From a cell’s mask at each timepoint, we subtract a mask that has been eroded by a disk of radius 5 to identify a 10-pixel-wide “rim” around the cell.
- Membrane sector intensity: By extending lines from the cell’s centroid in 10-degree increments, we subdivided the cell into 36 equal-angle regions. The intensity was then measured in a region formed by their intersection with the previously identified membrane region. The sector size was chosen because it was sufficiently small to be unlikely to contain multiple foci; doubling the number of sectors did not qualitatively change our results.
- Gβ-anchor correlation: To measure the extent of sequestration of Gβ to the ER at each timepoint, we computed the correlation of all cellular pixels (including both membrane and cytoplasm) between the Gβ and ER channels using each cell’s mask as described above.

### Identifying Cytoplasmic Oscillation

To identify which cells in a population were oscillating and characterize the timescale of oscillation, we turned to a Fourier approach (for the analyses of Figs 3 and 4). We found that the cytoplasmic LimE-GFP levels undergo strong, regular periodic fluctuations. From each cytoplasmic intensity timecourse, we subtracted a 30 s moving average to center cytoplasmic fluctuations on a mean value of zero (eliminating intensity fluctuations during cell movement or photobleaching) and computed the discrete Fourier transform of this mean-centered signal. Cells were then marked as “oscillating” if any sampling frequency between 0.05 and 0.2 Hz contained at least 10% of the cytoplasmic signal’s total power (see S4 Fig for oscillating and nonoscillating representative cells). These frequencies correspond to periods ranging from 5 to 20 s, which covered the range of frequencies we observed in a preliminary analysis across more than 50 oscillating cells. Each cell’s oscillation frequency was then taken to be the sampling frequency at which the power was maximal.

### Analysis of Dual Reporter Movies

To understand how cortical LimE dynamics relate to those of other cytoskeletal factors, we sought to correlate LimE-RFP with other reporters (GFP fusions to HSPC300, Coronin, the ABD actin binding domain of ABP120, and Arp2). To identify cells expressing both LimE and a second reporter, we thresholded cells using both GFP and RFP fluorescence. The cell’s cortex was identified as a 5-pixel-wide shell of this thresholded image for each cell. To compute the intensity of cytoskeletal foci around the cell’s cortex, we then subdivided the cortex into 36 equal-angle segments (sweeping out 10 degrees each) and measured the fluorescence intensity in both the GFP and RFP channels.

We then sought to compare the temporal dynamics of GFP and RFP in each spatial region from each cell. To do so, we calculated the cross-correlation between these two channels. For uncorrelated cytoskeletal factors (e.g., myosin, paxillin), we found that dynamics in GFP and RFP were uncorrelated, leading to a low-magnitude, flat cross-correlation. For correlated cytoskeletal factors (e.g., HSPC300, Coronin, Arp2, and the actin binding domain ABD), the cross-correlation peaked at the characteristic delay time between LimE and that particular cytoskeletal factor. We estimated this delay time by fitting a Gaussian distribution to the cross-correlation to identify the location of this peak—the resulting delay times are shown in Fig 5B.

### Measuring Polarization and Identifying Polarized and Unpolarized Time Periods

From the centroid and center of mass measurements described above, the direction and extent of polarization was determined by computing the vector between the center of mass ($\vec{c}$) and centroid ($\vec{n}$).

$$\vec{p}=\vec{c}-\vec{n}$$

The magnitude of $\vec{p}$ describes the extent of polarization, while its direction reflects the pole’s orientation.

We were also interested in identifying periods of time in which cells exhibit long-term, stable polarization (for the analyses of Fig 7). By inspecting many cell trajectories, we found that stable polarization was associated with a consistent direction of polarity—cells would retain a pole with a similar directional orientation, and changes in direction were associated with the formation of a new pole. Conversely, during unpolarized phases, fluctuations of actin around the membrane would lead to frequent changes in the direction of $\vec{p}$ (S14 Fig; lower panels). Thus, we implemented a greedy search algorithm to find continuous periods of time when the angle of polarization was contained in a 1-radian window and lasted at least 25 s, and measured the number and duration of these polar regions for each cell (S14 Fig shows two representative cells).

### Computing Hilbert Transform; Instantaneous Phase and Period

To assess the synchrony of oscillation between different membrane regions of a cell, we set out to measure each region’s oscillation phase at each timepoint. The phase of oscillation describes the current position of an oscillating signal on a sinusoidal curve (i.e., the rising or falling edge), and periodically rises from 0 to 2π. Thus, by comparing the phases between different regions of the membrane, we could assess whether they were oscillating in synchrony, with the phase rising and falling together, or whether at a single timepoint different membrane regions were at different points in their oscillating trajectories.

The analytic representation of a signal provided by the Hilbert transform is an ideal way to measure instantaneous properties of a signal containing periodic fluctuations such as the oscillation phase. For the time-varying LimE-GFP intensity in the n^th^ membrane sector *x*_*n*_ (*t*), the analytic signal

$${\tilde{x}}_{n}\left(t\right)=x_{n}\left(t\right)+i\;x_{n}\left(t\right)*\frac{1}{\pi t}$$

is a complex-valued function from which instantaneous properties of the signal’s oscillation can b e calculated, such as its instantaneous oscillation phase

$$\varphi_{n}\left(t\right)=∠\;{\tilde{x}}_{n}\left(t\right)$$

and frequency

$$\omega_{n}\left(t\right)={\dot{\varphi }}_{n}\left(t\right).$$

Phase measurement can be improved by first applying a low-pass filter to avoid noisy fluctuations from being interpreted as oscillation. Thus, we first applied a low-pass filter (an 8^th^ order Butterworth filter with a cutoff of 0.2 Hz) to each membrane trajectory before calculating its Hilbert transform, using custom Matlab code. We found this procedure to yield highly robust measurements of oscillation phase (S11 Fig) in both Gβ-sequestered and Gβ-functional cells. The instantaneous frequencies we measure from this approach are closely centered at ~10 s (S9 Fig) and are strikingly similar to those measured by Fourier analysis of cytoplasmic oscillation (Fig 3).

### Computing Synchrony

To assess synchrony between different membrane regions, we measured the breadth of spread in oscillation phase between them, at all timepoints during oscillation. We first computed the “group phase”—the vector sum of all regions’ individual phases, weighted identically.

$$\varphi_{g}\left(t\right)=∠\left(\sum_{n}e^{i\varphi_{n}\left(t\right)}\right)$$

We assessed synchrony by computing the phase difference between each membrane region and the group phase at each timepoint, and measured how broad this distribution is in oscillating Gβ-sequestered and nonoscillatory Gβ-functional cells (S12 Fig shows histograms of two representative cells).

### Speed Determination for Fig 7E

To characterize the migration of Gβ-sequestered and Gβ-unsequestered cells, we tracked individual cells during 10 min movies, where fluorescent images were acquired once per second. Cells were automatically segmented by thresholding the fluorescent channel, and the centroid of each cell was automatically determined at each timepoint. At least 28 cells were tracked in each condition. From each cell’s centroid data, we calculated the root-mean-squared displacement x_rms_ over time for each cell, choosing 300 distinct 5-min intervals for each cell during the 10 min movie. We fit the data to the simple diffusion model

$$x_{rms}^{2}=2dDt,$$

where *d* = 2 is the dimensionality, *D* is the diffusion constant, and *t* is the current time. From this model, we estimated the diffusion coefficient for each cell, and computed the *p*-value for a difference in diffusion coefficients between Gβ-sequestered and Gβ-unsequestered cells (Fig 7E).

### Modeling

#### Constructing a simple model of coupled oscillators and input sensing

To get some insight into how oscillator coupling can affect input sensing, we built a simple model incorporating the essential elements of this process. We reiterate that the goal of this model is not to provide a detailed account of the full biochemical network of either chemoattractant sensing or cell polarization, and a number of excellent models have already been published for both [5,69,92]. Rather, we are interested in whether we could construct a simple system to understand if coupling between individual oscillators can improve the system’s ability to entrain to an external input, and under what circumstances this may play an important role.

#### Modeling the cell membrane as a set of weakly coupled oscillators

Our model consists of *N* membrane domains arranged in a circle, each representing a single cytoskeletal oscillator. We assume each oscillator has a defined phase *θ*_*i*_, which progresses from 0 to 2π at a constant rate over one period. Each oscillator’s frequency *ω*_*i*_ is drawn randomly from a uniform distribution on the interval [*ω*_0_−*δω*, *ω*_0_+*δω*] (to account for this random sampling, each simulation was run at least 20 times, starting at different random initial conditions). Thus, the phase over time can be represented by the following expression:

$$\frac{d\theta_{i}}{dt}=\omega_{i}+\left(oscillator\;coupling\right)+\left(input\;coupling\right)$$

The following subsections will describe how we implement terms to account for oscillator-to-oscillator coupling and input-to-oscillator coupling.

#### Incorporating oscillator coupling

To implement coupling between oscillators, we assume that each oscillator *θ*_*j*_ has an effect on an oscillator *θ*_*i*_, speeding it up or slowing it down in proportion to the difference in their phase. We based this relationship on the well-known Kuramoto model of coupling in populations of oscillators [25,71].

$$\frac{d\theta_{i}}{dt}=\omega_{i}+\frac{k_{i}}{N}\text{sin}\left(\theta_{j}-\theta_{i}\right)$$

We also modeled an input source coupling to the first *M* oscillators. This input represents a localized source of activation, such as a spatially restricted source of chemoattractant.

We are interested in the effects of Gβ tuning the coupling between input-coupled oscillators (G protein-coupled receptors are activated locally upon chemoattractant binding, suggesting that Gβ could locally influence oscillator coupling). We therefore focused on one model in which the input sensing increases oscillator coupling: in this model, the coupling strength parameter is increased for all oscillators that receive an input stimulus. Thus,

$$k_{i}=\{\begin{array}{l} k_{1}+k_{2}\;\;\;\;\left(1\leq i\leq M\right)\\ k_{2\;}\;(M<i\leq N) \end{array}$$

#### Incorporating input coupling

We sought a simple way to implement oscillator coupling to an input, such that this coupling could be easily measured and is compatible with the modeling framework described above. We chose to describe the input as another oscillator that autonomously runs at a frequency $\omega_{IN}=\frac{1}{2}\omega_{0}$. The input frequency is therefore easily distinguishable from the natural frequencies of all membrane oscillators, and we can implement input coupling using the same mathematical term as for oscillator-to-oscillator coupling within the membrane.

Finally, we hypothesized that oscillator-to-oscillator synchrony might improve coupling in the case that input sensing is noisy—thus, sharing information between input-coupled oscillators may improve their ability to detect the input signal. To model input noise, we incorporated a single noise term *η*(*t*) in input-to-oscillator coupling. The noise function *η*(*t*) is drawn from a normal distribution at discrete sampling times (the sampling rate is chosen to be many times faster than the oscillation period). Taking into account all of these interactions, our final model can be represented as:

$$\frac{d\theta_{i}}{dt}=\{\begin{array}{l} \omega_{i}+\frac{\left(k_{1}+k_{2}\right)}{N}\text{sin}\left(\theta_{j}-\theta_{i}\right)\;+k_{IN}\text{sin}\left(\theta_{IN}-\theta_{i}+\eta \left(t\right)\right)\;\;\;\left(1\leq i\leq M\right)\\ \omega_{i}+\frac{k_{2}}{N}\text{sin}\left(\theta_{j}-\theta_{i}\right)\;\;\;\;\;\;\;\;\;\;\;\;\;\;\;\;\;\;\;\;\;\;\;\;\;\;\;\;\;\;\;\;\;\;\;\;\;\;\;\;\;\;\;\;\;\;\;\;\;\;\;\;\left(M<i\leq N\right) \end{array}$$

$$\eta \left[t_{k}\right]\;\sim N\left(0,\sigma \right)$$

#### Measuring synchrony

As a metric of synchrony, we reported what proportion of the total simulation time we observed input-coupled oscillators (membrane locations 1 to M) oscillating at a similar frequency to our input. Two oscillators were said to have similar frequencies when their frequencies differed by less than Δ*ω* = 0.1 rad/s, thus satisfying the inequality

$$\left|\frac{d\theta_{i}}{dt}\left(t\right)-\omega_{IN}\right|<\Delta \omega$$

As a control for our methodology, we also measured the input entrainment of oscillators without direct input-coupling terms (i.e., membrane locations M+1 to N). Broadly, we never observed substantial input coupling by these nonmembrane coupled regions, with coupling reported <10% of the time.

### Modeling Results

#### Strong oscillator-to-oscillator coupling leads to global, synchronized oscillation (S16 Fig)

We first tested whether increasing oscillator-to-oscillator coupling (represented by parameter *k*_*2*_) led to the expected increase in cell—cell coupling. This experiment is input-independent (the number of oscillators coupled to input, M, is set to 0), which renders other parameters (*k*_*1*_, *k*_*IN*_, *σ*) completely dispensable. Consistent with prior results describing the synchronization of weakly coupled oscillators as an abrupt phase transition, we found spontaneous large-scale synchrony emerge at a critical coupling strength of approximately *k*_2_ = 0.13 (S16 Fig). The parameter set used is shown in Table 1.

**Table 1:** Parameters used for varying oscillator-to-oscillator coupling.

Param	*ω*_0_	*δω*	*k*_1_	*k*_2_	*k*_*IN*_	*N*	*M*	σ
**Value**	0.5	0.2	N/A	*variable*	N/A	30	0	N/A

#### Oscillator-to-oscillator coupling does not increase sensitivity to weak, noise-free inputs (S21 Fig)

We next implemented input coupling to our membrane oscillators, with a coupling strength of *k*_*IN*_. We set out to understand how oscillator-to-input coupling and oscillator-to-oscillator coupling interact with one another, by varying the parameters (*k*_*1*_ and *k*_*IN*_, respectively) that determine their strength (see S21A Fig). We found that strong oscillator-to-input coupling (high *k*_*IN*_) drove complete frequency synchronization of membrane oscillators to the input, although with a constant phase lag (S21A and S21B Fig; upper panel).

Importantly, increased oscillator-to-oscillator coupling only served to weaken input sensing in this model (see the heat map of S21A Fig). At every value of *k*_*IN*_, increasing *k*_*1*_ decreased the fraction of time spent in an input-coupled state. Examining the behavior of the membrane oscillators at different values of *k*_*IN*_ and *k*_*1*_ lent insight into this observation (insets marked * and ^**o**^). For large *k*_*1*_, all membrane oscillators became input-insensitive and synchronized to one another at their natural frequency, similarly to the zero-input case (S16 Fig). This overall trend held for all parameter values tested. The parameter set shown in the supplement is in Table 2.

**Table 2:** Parameters used for simulating oscillator coupling to weak, noise-free inputs.

Param	*ω*_0_	*δω*	*k*_1_	*k*_2_	*k*_*IN*_	*N*	*M*	σ
**Value**	0.5	0.2	*variable*	0.1	*variable*	30	15	0

#### Oscillator-to-oscillator coupling can increase sensitivity to noisy inputs (Fig 9)

Are there any circumstances under which oscillator-to-oscillator coupling can improve input sensing? We hypothesized that coupling could provide a means of “sharing information” between membrane regions to better filter signal from noise in a noisy input (such as in the case of stochastic ligand-receptor binding within each local region of the plasma membrane). To test this hypothesis, we implemented a noise term in the model’s input coupling and varied oscillator-to-oscillator coupling to test for an increase or decrease in synchrony. The parameter set used is shown in Table 3.

**Table 3:** Parameters used for simulating oscillator coupling to noisy inputs.

Param	*ω*_0_	*δω*	*k*_1_	*k*_2_	*k*_*IN*_	*N*	*M*	σ
**Value**	0.5	0.2	*varies*	0.1	2	30	15	1.25

In contrast to the case of deterministic input sensing, noisy input sensing showed a clear benefit to intermediate levels of oscillator-to-oscillator coupling (Fig 9). For weak coupling, noise destroyed the ability for the membrane oscillators to lock onto the input (Fig 9, *k*_*1*_ = 0.1). For strong coupling, oscillators synchronized to each other, as seen for high coupling strength in our noise-free models (Fig 9, *k*_*1*_ = 3.5). However, for intermediate coupling strength, an enhancement of input sensing was clearly apparent, both from trajectories and from a metric assessing the fraction of time spent coupled to the input, which rose from approximately 50% to 75% (Fig 9, *k*_*1*_ = 2.5).

## Supporting Information

S1 Data Raw numerical values for all quantitative analyses in the main figures. Raw values from Figs 1B, 2A, 2B, 3C, 3D, 4A–4C, 5B–5E, 6B, 6C and 7C–7E. Data for figures are separated into different worksheets.
(XLSX)

S2 Data Raw numerical values for all quantitative analyses in the supplemental figures. Raw values from S1 and S4–S21 Figs.
(XLSX)

S1 Fig Chemoattractant-stimulated Ras and PIP_3_ responses are blocked after Rapamycin-mediated sequestration of Gβ. (A) Gβ-sequestration cells expressing the Ras activity (Ras*) reporter YFP-RBD(PI3K1) were incubated with rapamycin (1 μM; >20 min) and then stimulated with 100 μM folate. The plot shows the mean and standard deviation in cytoplasmic reporter intensity of individual unsequestered (*n* = 15) and Gβ-sequestered cells (*n* = 12), pooled from several stimulation experiments. (B) Gβ-sequestration cells expressing the PIP_3_ reporter PhdA-GFP were incubated with rapamycin (1 μM; >20 min) and stimulated with 100 μM folate. Plots show the mean and standard deviation in cytoplasmic reporter intensity of individual unsequestered (*n* = 9) and Gβ-sequestered cells (*n* = 17) pooled from several stimulation experiments. Raw data can be found in S2 Data.
(TIF)

S2 Fig Gβ-sequestration induces F-actin oscillations at the cortex. Strong LimE-GFP oscillations are apparent at the cortex in Gβ-sequestered cells. A confocal slice from the middle of a cell is stacked into a kymograph (t-stack; as in Fig 7A and 7B). In this representation, bright rings of LimE-GFP are apparent in Gβ-sequestered (+RAP) but not Gβ-unsequestered (-RAP) cells. Scale bar = 5 μm.
(TIF)

S3 Fig The origin of F-actin oscillations. (A) LimE-GFP accumulation at the cortex is not restricted to areas where Gβ remains in close proximity to the plasma membrane after rapamycin addition. The red circles indicate areas where no Gβ is apparent, yet LimE-GFP is strongly localized during oscillations. Scale bar = 5 μm. (B) LimE oscillations are not recapitulated by bringing the ER in touch with the plasma membrane. The ER was recruited to the cAMP receptor (DH1:cAR1-RFP-FRB; calexinA-CFP-FKBP; LimE-GFP) or a myristoylation tag (Ax2: myr(SRC)-YFP-FRB; calnexinA-CFP-FKBP; LimE-RFP), and the percentage of cells with LimE oscillations was determined. Cells from at least 2 d are combined. The image panels show examples of cAR1 and myr(SRC) before and after treatment with rapamycin. Scale bar = 5 μm.
(TIF)

S4 Fig A computational pipeline to analyze F-actin oscillations. (A) Schematic of data processing steps to assess cytoplasmic LimE-GFP oscillations. Slow fluctuations in mean intensity were removed from each single-cell cytoplasmic trajectory by subtracting a 30 s moving average. The Fourier transform for each trajectory was then computed and normalized to the same total signal power to account for differences in reporter expression level and oscillation amplitude. When a single frequency peak contained more than 10% of the total signal power, a trajectory was considered oscillating. The peak frequency was also measured. (B) Representative single-cell trajectories for an oscillating cell (left) and nonoscillating cell (right), showing both time-domain (upper plot) and frequency-domain representations (lower plot). The 10% threshold is shown (solid black line). (C) Histogram of oscillation period across >75 oscillating, rapamycin-treated cells. Raw data can be found in S2 Data.
(TIF)

S5 Fig Gβ-sequestration can induce rapid waves of actin polymerization that travel around the cell perimeter. After Gβ sequestration, rotating waves of LimE-GFP traveling around the cell periphery are observed in some cells (5/46 cells). This behavior is not seen in unsequestered cells (0/28 cells). Scale bar = 5 μm. Numbers indicate time in seconds after start of recording. Raw data can be found in S2 Data.
(TIF)

S6 Fig Global oscillations of F-actin formation start rapidly after sequestration of Gβ. Top panels show a confocal slice over time for a cell expressing the Gβ sequestration system and LimE-GFP before (grey) and after (orange) addition of 1 μM rapamycin (S3 Movie shows entire sequence). Oscillations of LimE (arbitrary units) are observed within 40 s of rapamycin addition. Periods of oscillation are interrupted by periods without oscillation (see text and Fig 7A and 7B). Scale bar = 5 μm. Numbers indicate time in seconds after start of recording. Rapamycin is added at *t* = 300 s. Raw data can be found in S2 Data.
(TIF)

S7 Fig Extent of Gβ-sequestration can be titrated by competing rapamycin with FK506. (A) Schematic showing the competing effects of rapamycin and FK506. While rapamycin mediates heterodimerization of FRB and FKBP, FK506 acts as a competitive inhibitor for this heterodimerization. (B) Increasing the amount of Gβ sequestration (by decreasing the concentration of the competitive inhibitor FK506 [green box; *x*-axis]) increases the percentage of oscillating cells. The oscillating cells (orange dots) and nonoscillating cells (black dots) make up the histogram shown on top (histogram; *x*-axis). By inspecting the data horizontally, it is apparent that cells with a higher extent of sequestration (green box; *y*-axis) are more likely to oscillate (histogram; *y*-axis). The *y*-axes of this figure recapitulate Fig 4A in the main text. Larger effective rapamycin concentrations lead to a higher extent of Gβ sequestration, which makes cells more likely to oscillate. Raw data can be found in S2 Data.
(TIF)

S8 Fig Amplitude of oscillations do not change with Gβ sequestration level. A higher level of Gβ sequestration (a lower concentration of active Gβ) does not affect the amplitude of LimE-GFP oscillations. Cells and treatment conditions are the same as analyzed in Fig 4A and 4B; (*n* ≥ 20 cells per sequestration bin; plotted are means +/- stdev). Raw data can be found in S2 Data.
(TIF)

S9 Fig The period of F-actin oscillations in individual sectors. Histograms of oscillation periods collected from all timepoints and membrane sectors of a representative cell show peaks at ~10 s in all three conditions. The green curve falls on top of the blue curve for most of the histogram. Raw data can be found in S2 Data.
(TIF)

S10 Fig Dual color observation of F-actin and its regulators at foci. (A) Plot of the intensity of both LimE-RFP and Coronin-GFP at the same peripheral spot over time. The focal spot shown in the traces is circled in the still images. The time interval that corresponds to the still images is further indicated with grey overlay on the traces. (B) The correlation between individual traces was computed. By fitting a Gaussian curve to the data, the delay times shown in Fig 5B could be determined. Plotted are means +/- SEM. Raw data can be found in S2 Data.
(TIF)

S11 Fig The Hilbert transform applied to determine phase information from oscillating actin foci on the cell’s cortex. Shown are timecourses of LimE-GFP intensity from representative membrane sectors (top graphs) and the Hilbert-extracted phase information (bottom graphs) for Gβ-null, Gβ-unsequestered, and Gβ-sequestered cells (data from one cell for each condition are shown). In each case, the phase increases from -2π to 2π during each membrane LimE-GFP pulse. Raw data can be found in S2 Data.
(TIF)

S12 Fig Extent of synchrony between membrane regions differs between oscillating and nonoscillating cells. Histograms of the phase difference between each membrane sector and the “group phase” (the mean response of all membrane sectors) is shown across all sectors and timepoints for two representative cells. Cells undergoing whole-field oscillation (right panel) are more synchronous, displaying a tighter clustering around the group phase than nonoscillatory cells (left panel). It is worth noting that membrane sectors in nonoscillatory cells show weak coupling, and membrane sectors in oscillatory cells do not completely phase lock. Raw data can be found in S2 Data.
(TIF)

S13 Fig Membership of oscillators in the phase-locked group is fluid. During a 50-s window, the top 25% most-synchronized membrane regions (blue) and least-synchronized membrane regions (red) of one Gβ-sequestered cell were determined (left). Following one blue sector and one red sector over time shows that, at different times, either sector can be “in sync” and “out of sync” with the periphery average. Raw data can be found in S2 Data.
(TIF)

S14 Fig Stability of polarization angle as well as the decrease in cytoplasmic LimE-GFP intensity are good classifiers of cell polarity. Cytoplasmic LimE-GFP intensity (upper panels) and polarization angle (lower panels) are shown for two cells expressing the Gβ-sequestration system—one Gβ-sequestered, oscillatory cell (left) and one Gβ-unsequestered, nonoscillatory cell (right). Periods of stable cell polarity are indicated in gray. Images of the cells during polarized and nonpolarized phases (below) show that a decrease in cytoplasmic intensity and the stability of pole angle are tightly correlated with the appearance of polarized regions of cortical LimE-GFP. Raw data can be found in S2 Data.
(TIF)

S15 Fig Strength of polarity is similar between Gβ-sequestered and unsequestered cells. The magnitude of the vector M is plotted for individual cells during polarized and nonpolarized phases. A similar area of the diagram is occupied during polarized phases for both Gβ-sequestered and Gβ-unsequestered cells. Raw data can be found in S2 Data.
(TIF)

S16 Fig Increasing oscillator-to-oscillator coupling drives a phase transition to synchrony. In the absence of external input, coupling in our model is only determined by parameter *k*_2_. Varying its magnitude leads to an abrupt phase transition from weakly coupled oscillators to large-scale synchrony. This recapitulates the phenotype we observe after Gβ-sequestration. The group behavior of the oscillators for two values of *k*_*2*_ is shown. Raw data can be found in S2 Data.
(TIF)

S17 Fig Gβ-sequestration increases the fraction of sectors that contain an actin oscillator. (A) Plot shows the fraction of membrane sectors containing an oscillating actin focus. We used the same dataset as in Fig 5E (Gβ-null [mutants]; Gβ+ [Gβ-unsequestered]; and Gβ-sequestered cells displaying whole-field oscillations). For whole-field oscillating cells, a higher fraction of membrane sectors contains an actin oscillator. (B) Images show a maximum intensity projection of 30 frames (1 frame/second) of a representative cell for which Gβ sequestration induces global oscillations. Cell is shown before and after the perturbation. Graphs show the fraction of membrane sectors, or the amplitude of the membrane sectors, that oscillate for four sequestered cells before and after rapamycin-mediated sequestration. Overall, amplitude is unchanged, while the fraction of oscillating sectors increases. For cell 2, all membrane sectors oscillate, although not in phase with one another, prior to Gβ sequestration. Raw data can be found in S2 Data.
(TIF)

S18 Fig Oscillating actin foci are suppressed during cell polarization in wild-type cells. (A) The top panels show representative images of one Gβ-unsequestered cell over time as the cell forms a pole of high LimE-GFP intensity (*t* = 180–280 s). Bottom panels show individual intensity traces and mean intensity of all sectors not contributing to the pole. As the cell polarizes, actin foci at the periphery are lost (see also S6 Movie) (B) LimE-GFP intensity of actin foci at the periphery does not decrease because of competition with a pole. Maximum LimE-GFP intensity of individual actin structures appearing in the middle of the cell was quantified and plotted as a function of time of their appearance. Structures, most likely endocytic patches, are colored differently based on their appearance relative to a cell polarization event (gray): before polarization (blue), during polarization (green), and after polarization (red). All data are from the representative cell pictured in (A). (C) Quantitation of the peak intensity from each actin trajectory in (B). Raw data can be found in S2 Data.
(TIF)

S19 Fig pp242 as an inhibitor of TORC2 function in Dictyostelium cells. 2x10^7^ cells/ml in DB buffer were incubated with the indicated concentrations of pp242 or the DMSO solvent only. At time 0, cells were stimulated with 50 mM folic acid or buffer (vehicle control), and samples were collected and blotted for phosphorylation at T309 of PKBR1. A dose-dependent reduction in peak levels (*t* = 15 s) is apparent.
(TIF)

S20 Fig Mean squared displacement increases linearly with time. Plot of the mean squared displacement (mean +/- SEM) as a function of time for two representative cells, one for which Gβ has been sequestered and one for which Gβ has not been sequestered. Cells were automatically tracked by their centroid from thresholded fluorescent images taken each second (same data from which numbers for Fig 7C and 7D were extracted). Each cell’s mean squared displacement was calculated from all trajectories during a 5-min interval of a 10-min movie. Best-fit line (proportional to the estimated diffusion coefficient) is shown in blue. Raw data can be found in S2 Data.
(TIF)

S21 Fig Oscillator-to-oscillator coupling does not improve local sensing of weak inputs. (A) We kept *k*_*2*_ at a fixed, low value (0.1) and addressed how oscillator-to-oscillator coupling in an area experiencing an input (*k*_*1*_) affected sensing of inputs (*k*_*IN*_). High values of *k*_*IN*_ lead to perfect synchronization of input and oscillator dynamics (input sensing). Increasing *k*_*1*_ only decreases the extent of synchronization (see heat map and graph). (B) Traces of indicated, representative areas of the heat map in (A) are shown. Raw data can be found in S2 Data.
(TIF)

S1 Movie Rapamycin-induced Gβ sequestration. The Gβ sequestration strain (Gβ-: FRB-RFP-Gβ, calnexinA-YFP-FKBP) is challenged with 1 μM rapamycin at the start of the movie. Sequestration of Gβ can be seen by the increased colocalization of RFP and YFP. Recorded at 1 frame/min; played at 10 frames/second.
(AVI)

S2 Movie Electrotaxis of Gβ-sequestered cells. Movie corresponds to still images in Fig 2B. Cells were recorded at 2 frames/min in the presence of an electric field and rapamycin. Red label: “wt” (Gβ+/anchor-); green label: “Gβ-null”(Gβ-/anchor-); yellow label: “Gβ-sequestered” (Gβ+/anchor+).
(AVI)

S3 Movie Onset of oscillations after rapamycin-induced Gβ sequestration. Most behaviors described in this paper are apparent in this movie of the Gβ sequestration strain expressing the F-actin binding probe LimE-GFP. The movie is split (LimE-GFP on the left; FRB-RFP-Gβ on the right). After frame 300, addition of 1 μM rapamycin (labeled) induces sequestration of Gβ. This starts to show as visible clustering of Gβ about halfway through the movie. Within 40 s, LimE-GFP begins rapid whole-field oscillations that alternate with polarized accumulation of F-actin. Toward the end of the movie, circular rotations of LimE-GFP can be observed. The movie was recorded at 1 frame/second and is played back at 30 frames/second. Portions of this movie correspond to S9 Fig.
(MOV)

S4 Movie Kymograph (t-stack) of actin accumulation in a Gβ unsequestered cell (- rapamycin). 360-degree rotation of a t-stack showing F-actin accumulation at the cortex (visualized with LimE-GFP) during polar and apolar phases. Movie corresponds to Fig 7A and is played at 72 frames/second. Vertical axis represents time (first time point on top, last time point on bottom).
(MOV)

S5 Movie Kymograph (t-stack) of actin accumulation in a Gβ-sequestered cell (+ rapamycin). 360-degree rotation of a t-stack showing F actin accumulation at the cortex (visualized with LimE-GFP) during polar and apolar phases. Movie corresponds to Fig 7B and is played at 72 frames/second. Vertical axis represents time (first time point on top, last time point on bottom).
(MOV)

S6 Movie Actin oscillators during polarization. Movie corresponds to the cell in S23 Fig, imaged in the coverslip plane during a cell polarization event. LimE-GFP reports oscillators and filamentous actin accumulation.
(AVI)

## Abbreviations

cAMP: cyclic-AMP
ER: endoplasmic reticulum
GPCR: G-protein-coupled receptor
NPF: nucleation promoting factor
PIP_3_: phosphatidylinositol 3,4,5-trisphosphate
WASP: Wiskott-Aldrich Syndrome Protein
wt: wild-type
