# Supplementary figures and images for: Gβ Regulates Coupling between Actin Oscillators for Cell Polarity and Directional Migration
Source: PLoS Biol. 2016 Feb 18;14(2):e1002381. doi: 10.1371/journal.pbio.1002381 (PMC4758609; doi:10.1371/journal.pbio.1002381)

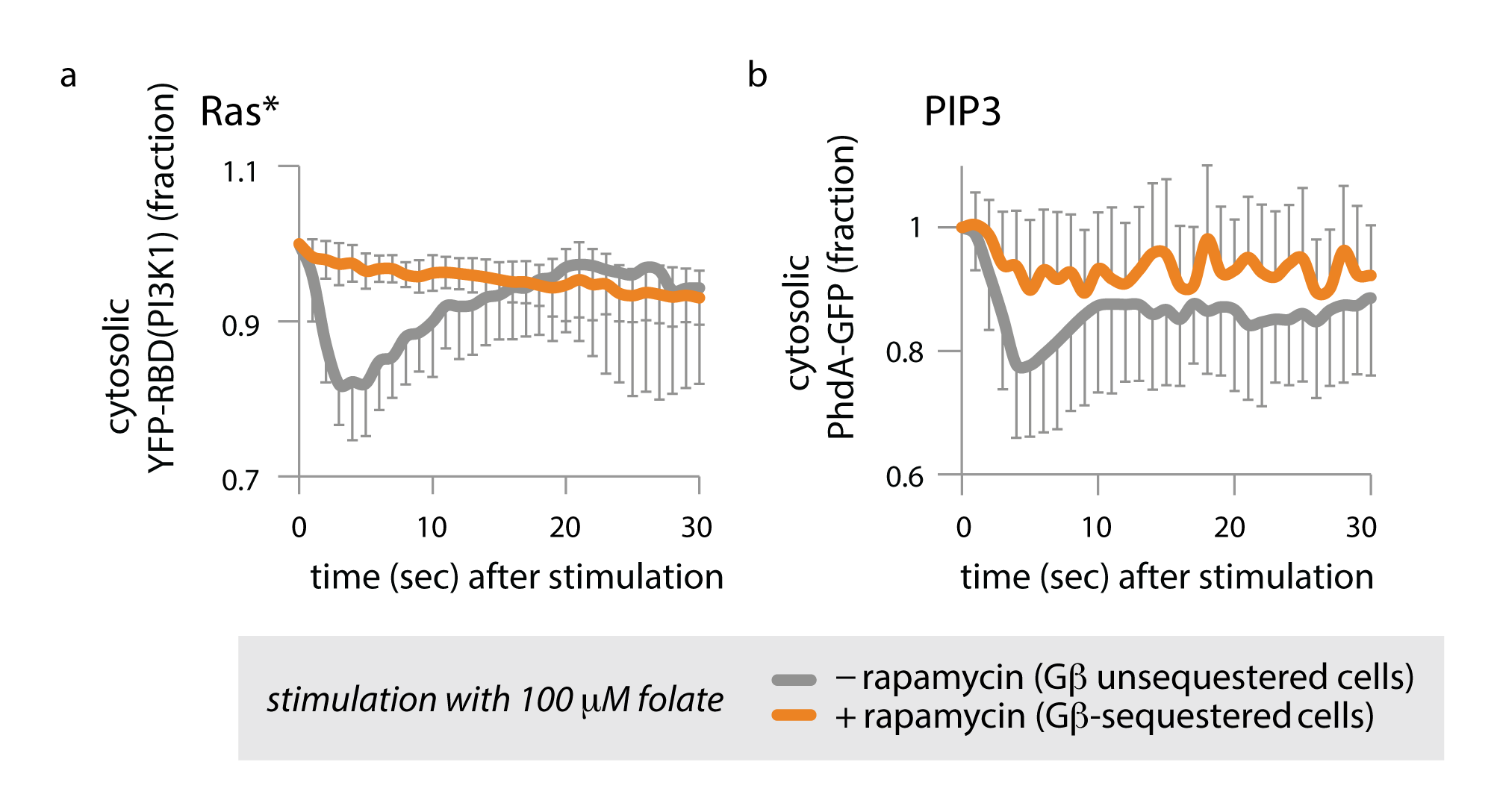

Supplement: S1 Fig — (A) Gβ-sequestration cells expressing the Ras activity (Ras*) reporter YFP-RBD(PI3K1) were incubated with rapamycin (1 μM; >20 min) and then stimulated with 100 μM folate. The plot shows the mean and standard deviation in cytoplasmic reporter intensity of individual unsequestered (n = 15) and Gβ-sequestered cells (n = 12), pooled from several stimulation experiments. (B) Gβ-sequestration cells expressing the PIP3 reporter PhdA-GFP were incubated with rapamycin (1 μM; >20 min) and stimulated with 100 μM folate. Plots show the mean and standard deviation in cytoplasmic reporter intensity of individual unsequestered (n = 9) and Gβ-sequestered cells (n = 17) pooled from several stimulation experiments. Raw data can be found in S2 Data. (TIF) [file pbio.1002381.s003.tif]

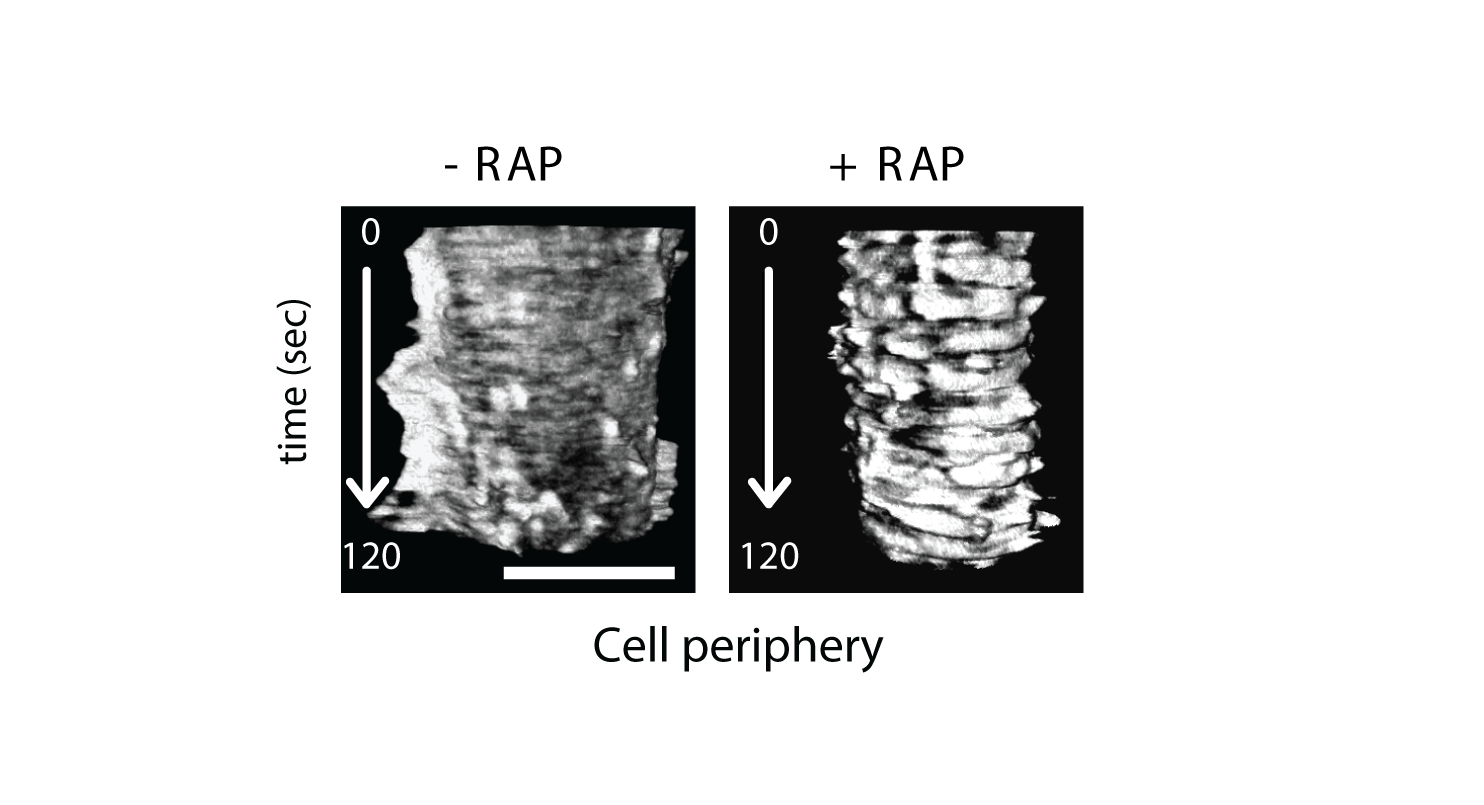

Supplement: S2 Fig — Strong LimE-GFP oscillations are apparent at the cortex in Gβ-sequestered cells. A confocal slice from the middle of a cell is stacked into a kymograph (t-stack; as in Fig 7A and 7B). In this representation, bright rings of LimE-GFP are apparent in Gβ-sequestered (+RAP) but not Gβ-unsequestered (-RAP) cells. Scale bar = 5 μm. (TIF) [file pbio.1002381.s004.tif]

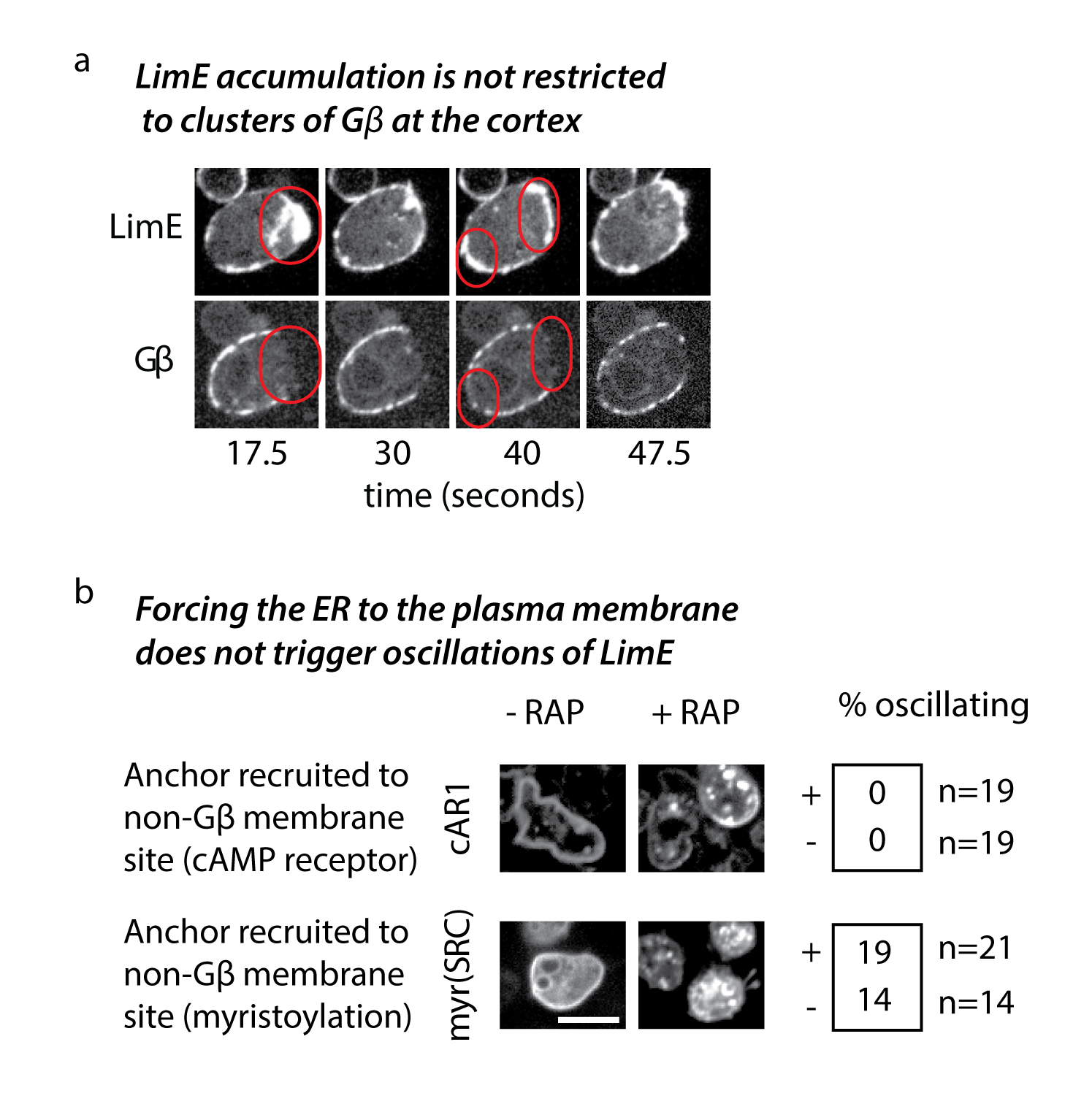

Supplement: S3 Fig — (A) LimE-GFP accumulation at the cortex is not restricted to areas where Gβ remains in close proximity to the plasma membrane after rapamycin addition. The red circles indicate areas where no Gβ is apparent, yet LimE-GFP is strongly localized during oscillations. Scale bar = 5 μm. (B) LimE oscillations are not recapitulated by bringing the ER in touch with the plasma membrane. The ER was recruited to the cAMP receptor (DH1:cAR1-RFP-FRB; calexinA-CFP-FKBP; LimE-GFP) or a myristoylation tag (Ax2: myr(SRC)-YFP-FRB; calnexinA-CFP-FKBP; LimE-RFP), and the percentage of cells with LimE oscillations was determined. Cells from at least 2 d are combined. The image panels show examples of cAR1 and myr(SRC) before and after treatment with rapamycin. Scale bar = 5 μm. (TIF) [file pbio.1002381.s005.tif]

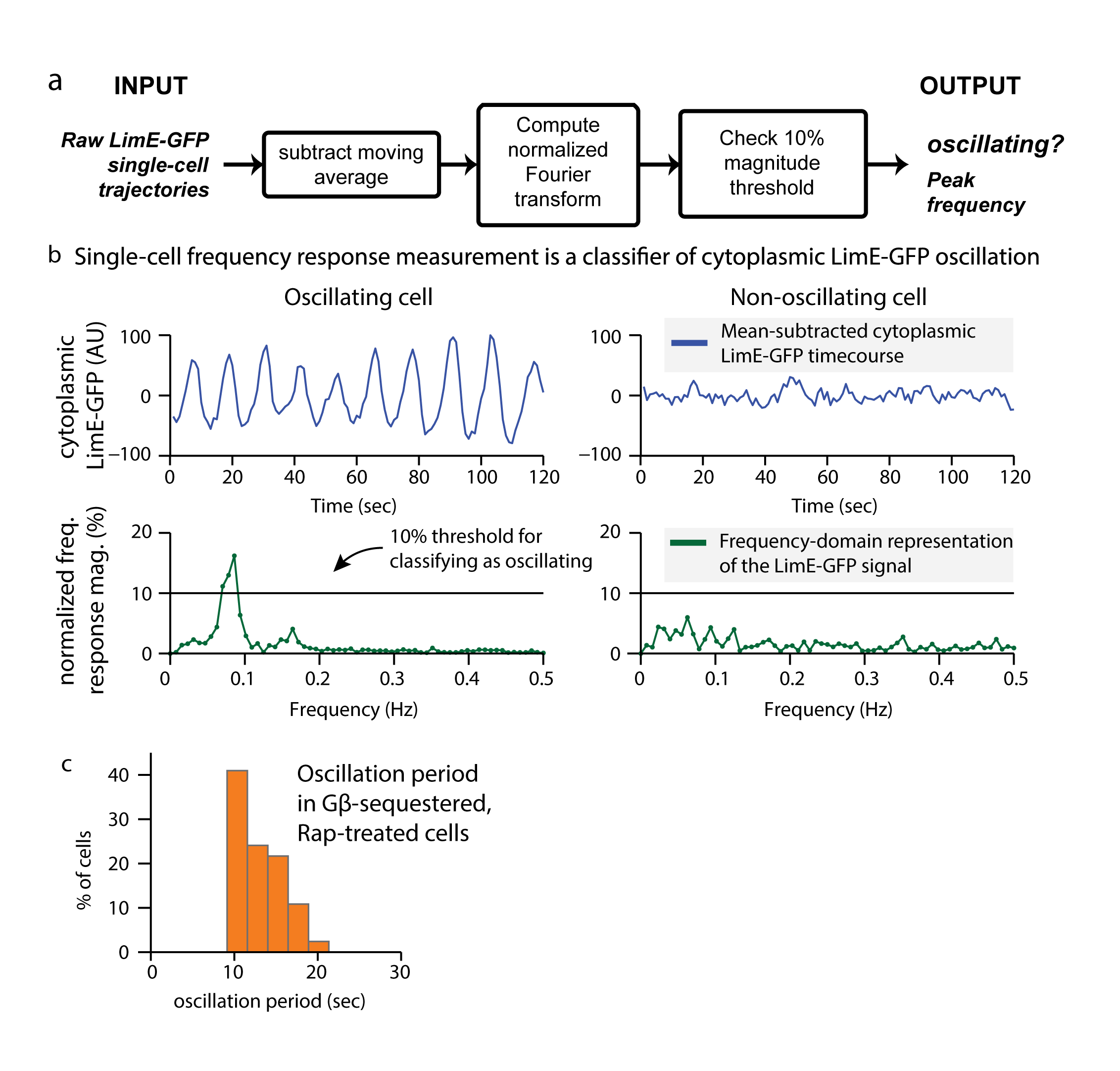

Supplement: S4 Fig — (A) Schematic of data processing steps to assess cytoplasmic LimE-GFP oscillations. Slow fluctuations in mean intensity were removed from each single-cell cytoplasmic trajectory by subtracting a 30 s moving average. The Fourier transform for each trajectory was then computed and normalized to the same total signal power to account for differences in reporter expression level and oscillation amplitude. When a single frequency peak contained more than 10% of the total signal power, a trajectory was considered oscillating. The peak frequency was also measured. (B) Representative single-cell trajectories for an oscillating cell (left) and nonoscillating cell (right), showing both time-domain (upper plot) and frequency-domain representations (lower plot). The 10% threshold is shown (solid black line). (C) Histogram of oscillation period across >75 oscillating, rapamycin-treated cells. Raw data can be found in S2 Data. (TIF) [file pbio.1002381.s006.tif]

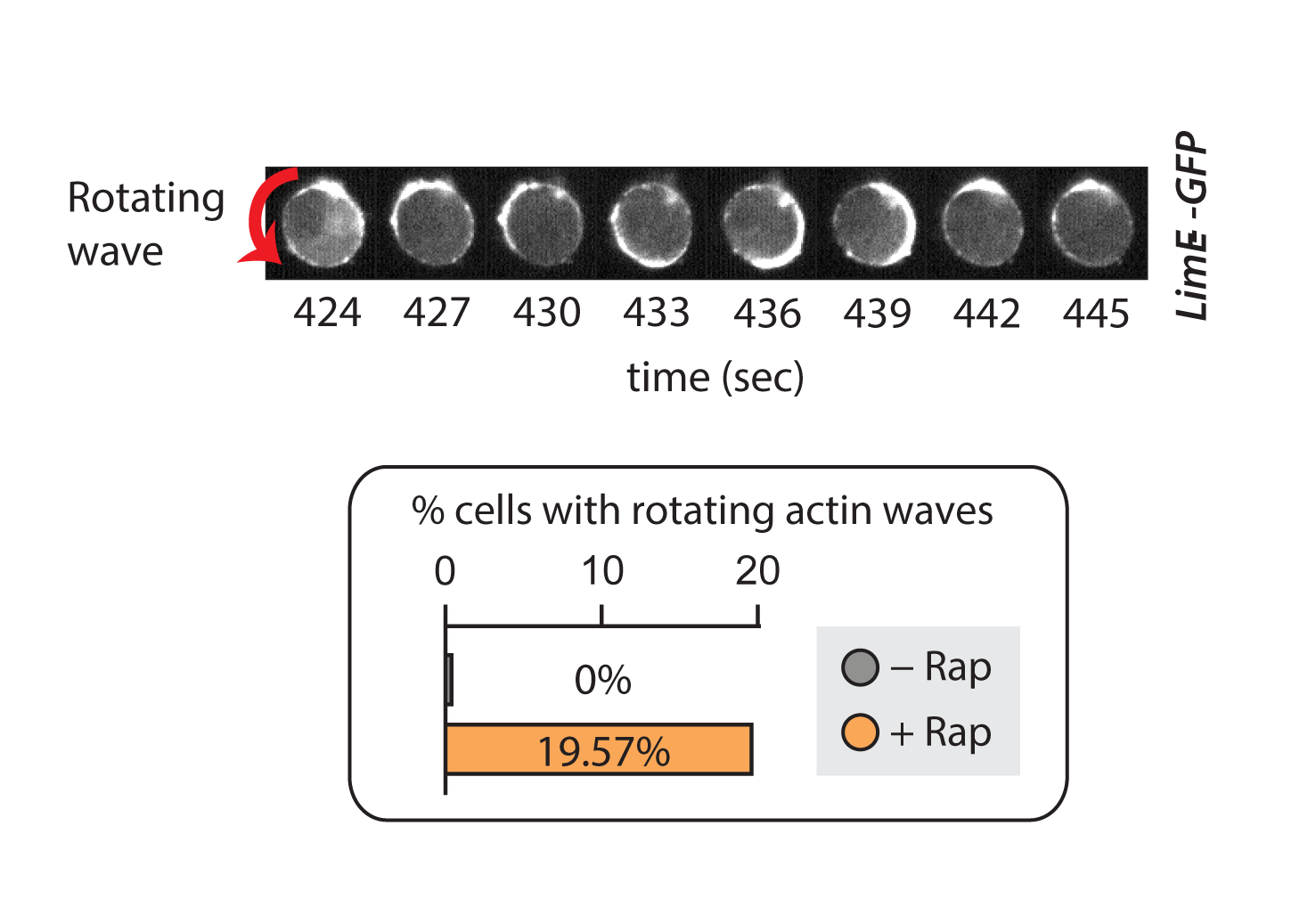

Supplement: S5 Fig — After Gβ sequestration, rotating waves of LimE-GFP traveling around the cell periphery are observed in some cells (5/46 cells). This behavior is not seen in unsequestered cells (0/28 cells). Scale bar = 5 μm. Numbers indicate time in seconds after start of recording. Raw data can be found in S2 Data. (TIF) [file pbio.1002381.s007.tif]

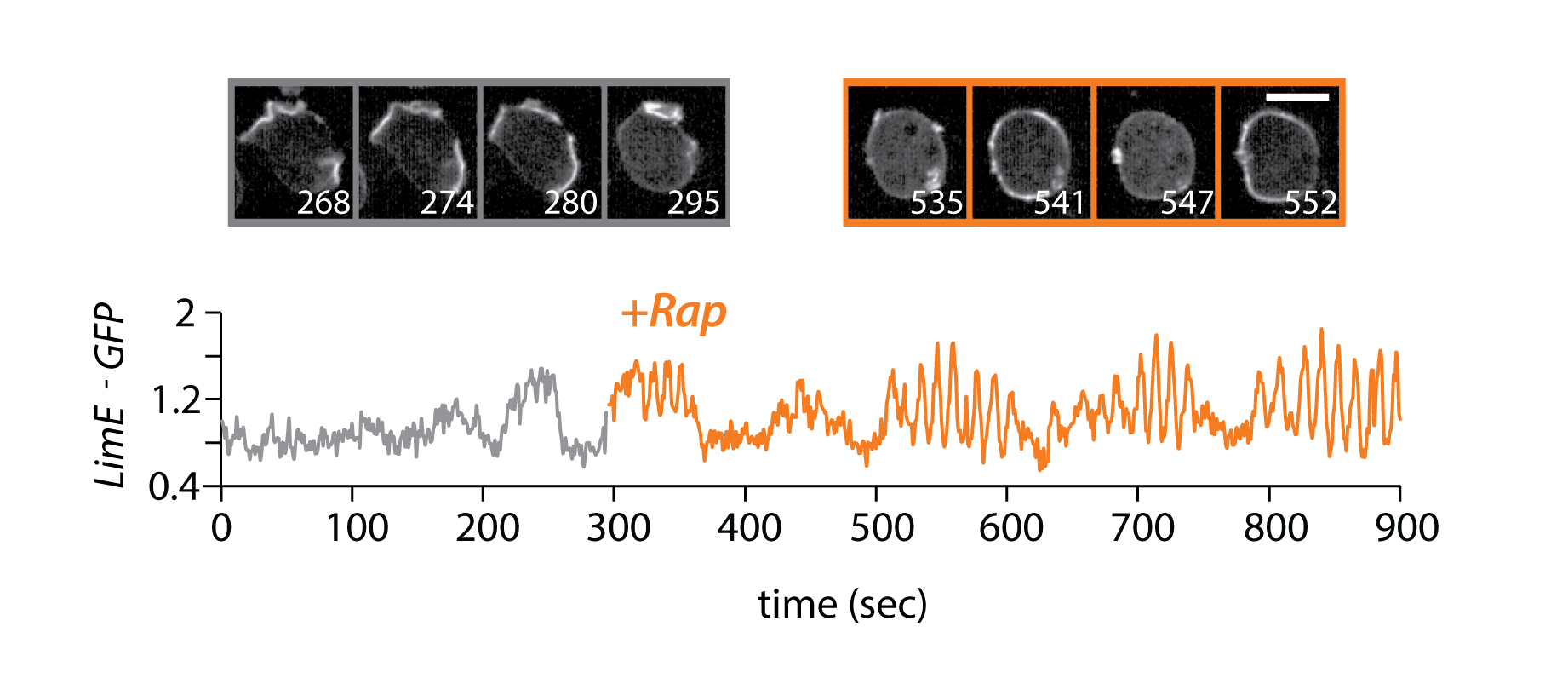

Supplement: S6 Fig — Top panels show a confocal slice over time for a cell expressing the Gβ sequestration system and LimE-GFP before (grey) and after (orange) addition of 1 μM rapamycin (S3 Movie shows entire sequence). Oscillations of LimE (arbitrary units) are observed within 40 s of rapamycin addition. Periods of oscillation are interrupted by periods without oscillation (see text and Fig 7A and 7B). Scale bar = 5 μm. Numbers indicate time in seconds after start of recording. Rapamycin is added at t = 300 s. Raw data can be found in S2 Data. (TIF) [file pbio.1002381.s008.tif]

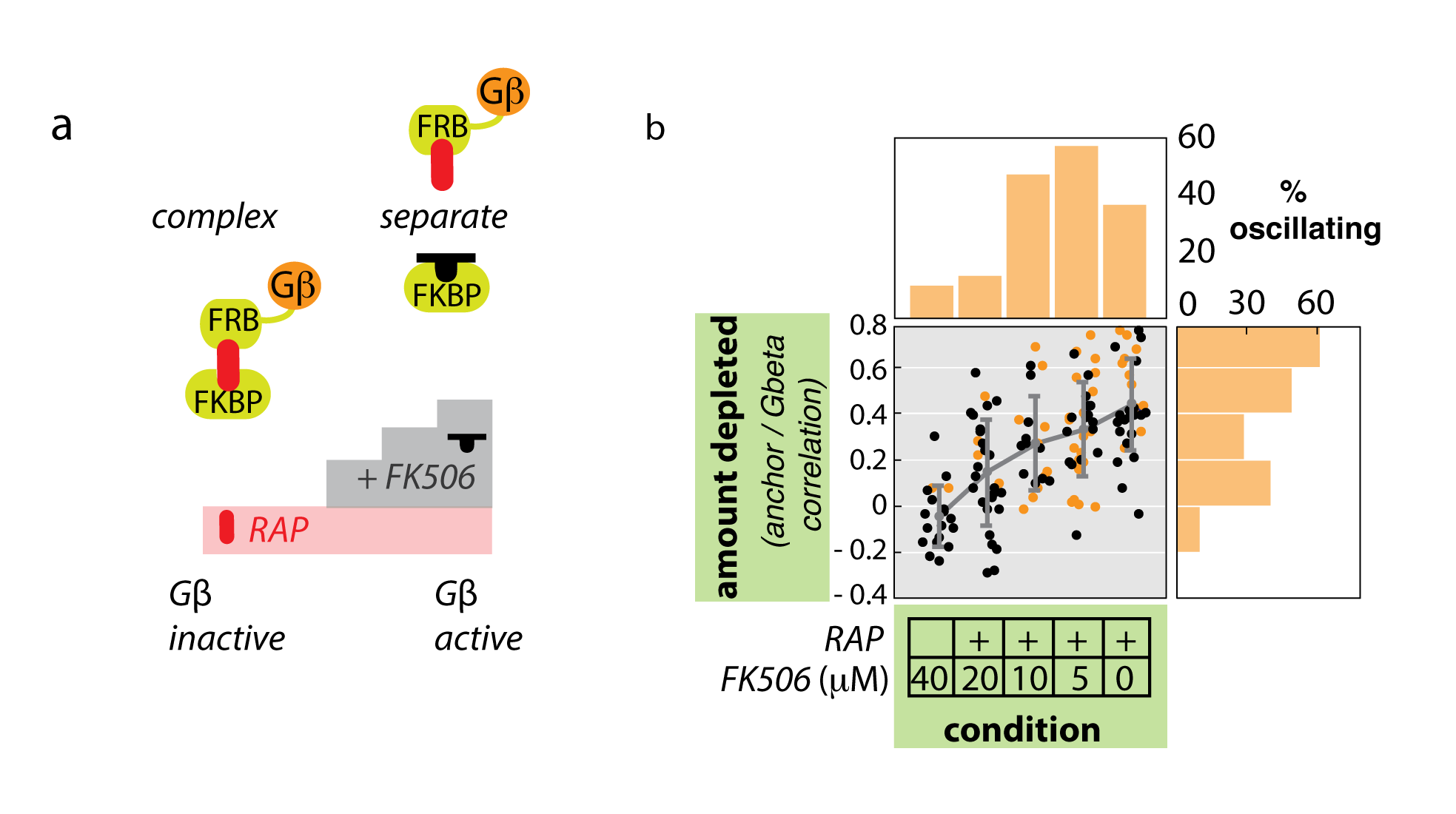

Supplement: S7 Fig — (A) Schematic showing the competing effects of rapamycin and FK506. While rapamycin mediates heterodimerization of FRB and FKBP, FK506 acts as a competitive inhibitor for this heterodimerization. (B) Increasing the amount of Gβ sequestration (by decreasing the concentration of the competitive inhibitor FK506 [green box; x-axis]) increases the percentage of oscillating cells. The oscillating cells (orange dots) and nonoscillating cells (black dots) make up the histogram shown on top (histogram; x-axis). By inspecting the data horizontally, it is apparent that cells with a higher extent of sequestration (green box; y-axis) are more likely to oscillate (histogram; y-axis). The y-axes of this figure recapitulate Fig 4A in the main text. Larger effective rapamycin concentrations lead to a higher extent of Gβ sequestration, which makes cells more likely to oscillate. Raw data can be found in S2 Data. (TIF) [file pbio.1002381.s009.tif]

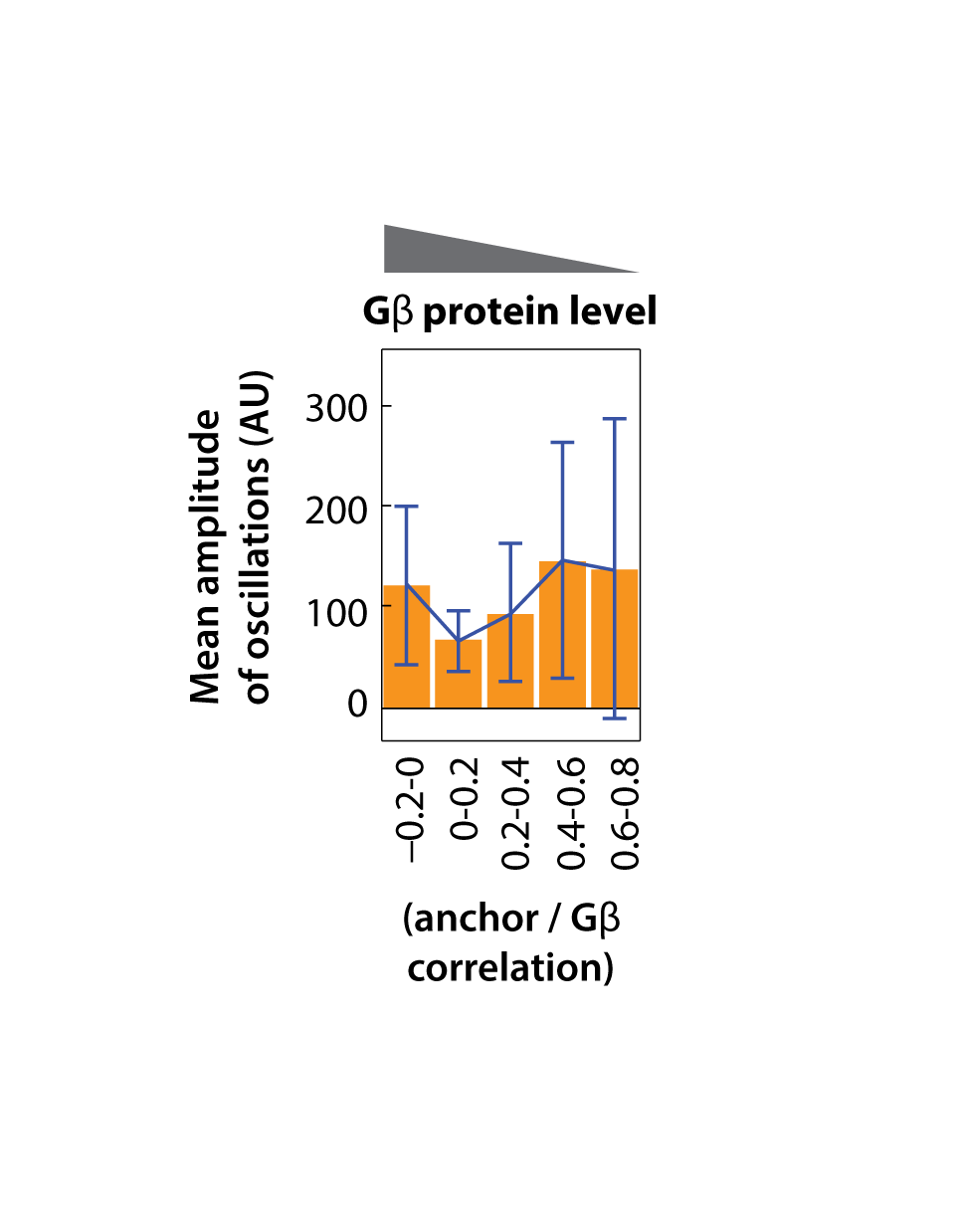

Supplement: S8 Fig — A higher level of Gβ sequestration (a lower concentration of active Gβ) does not affect the amplitude of LimE-GFP oscillations. Cells and treatment conditions are the same as analyzed in Fig 4A and 4B; (n ≥ 20 cells per sequestration bin; plotted are means +/- stdev). Raw data can be found in S2 Data. (TIF) [file pbio.1002381.s010.tif]

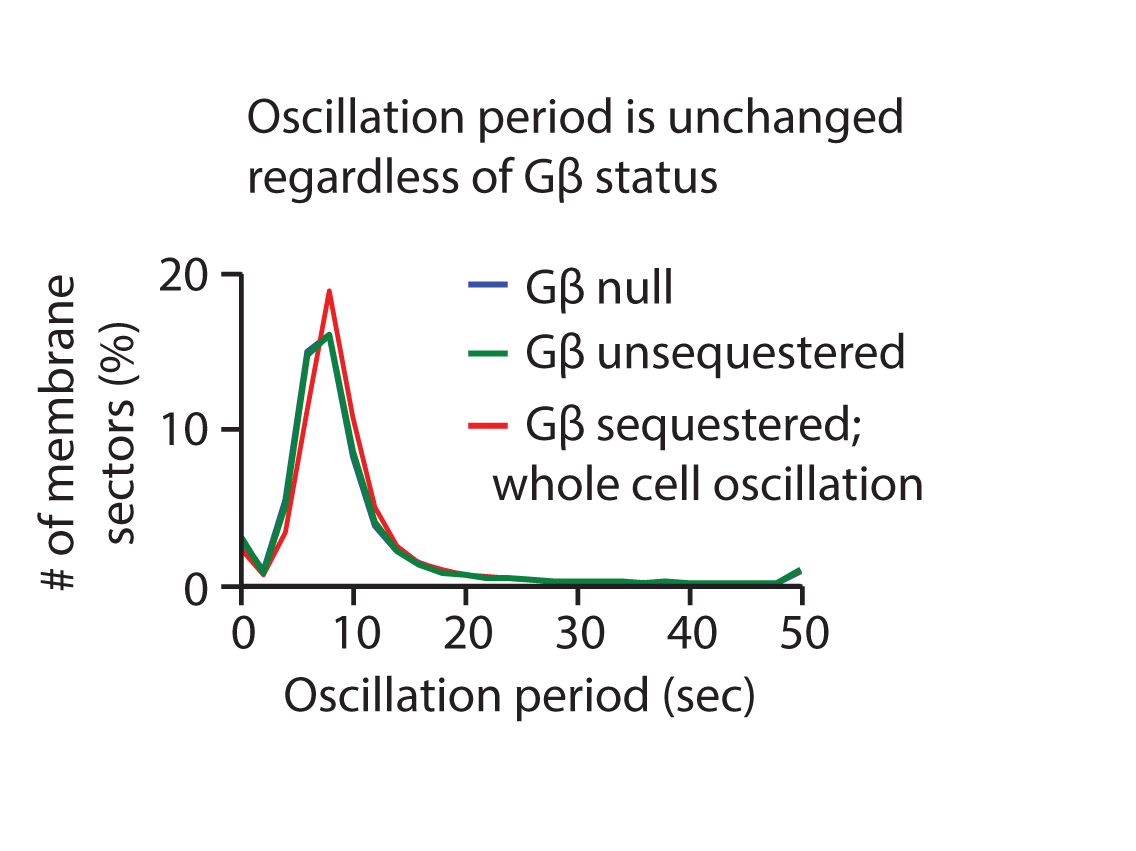

Supplement: S9 Fig — Histograms of oscillation periods collected from all timepoints and membrane sectors of a representative cell show peaks at ~10 s in all three conditions. The green curve falls on top of the blue curve for most of the histogram. Raw data can be found in S2 Data. (TIF) [file pbio.1002381.s011.tif]

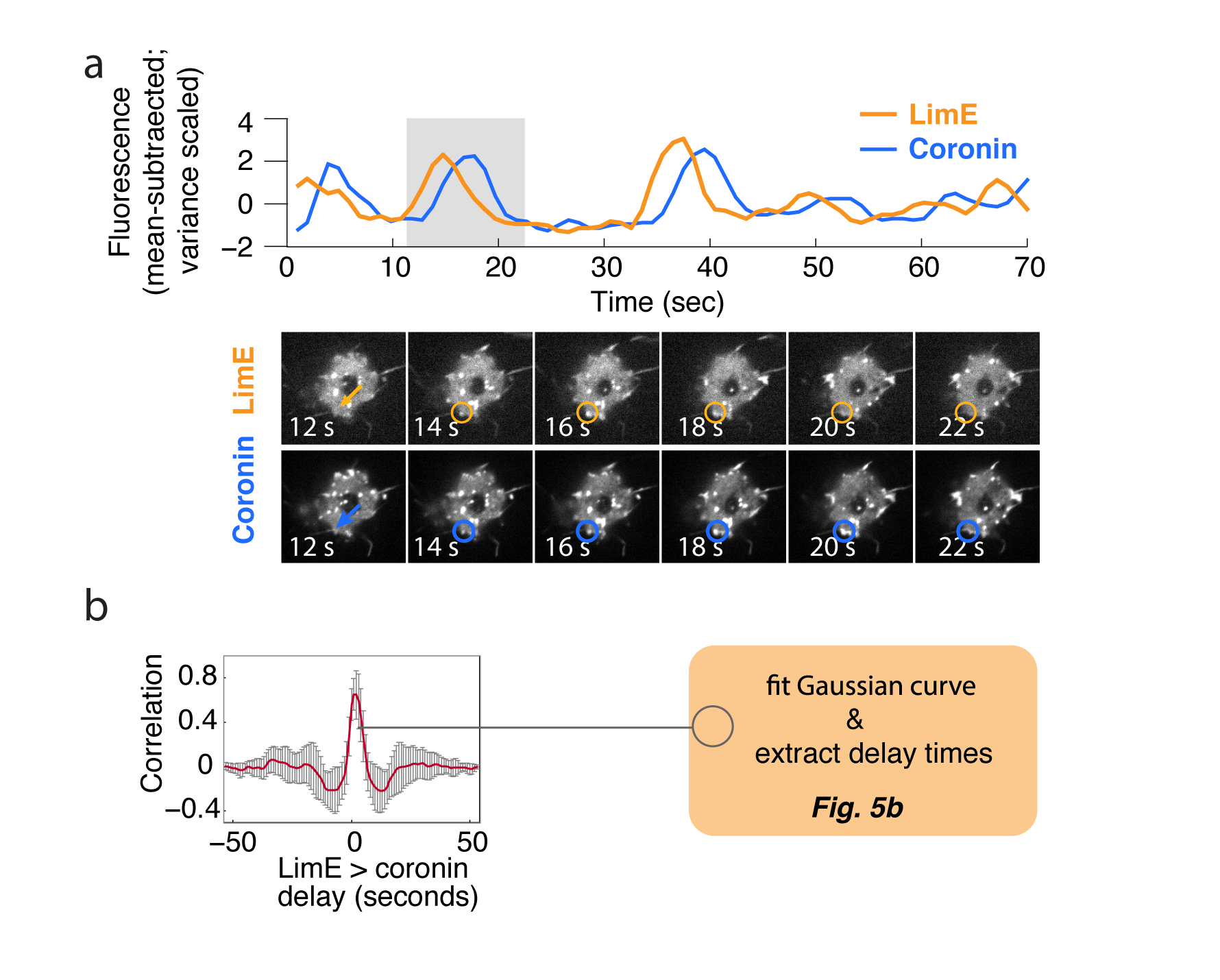

Supplement: S10 Fig — (A) Plot of the intensity of both LimE-RFP and Coronin-GFP at the same peripheral spot over time. The focal spot shown in the traces is circled in the still images. The time interval that corresponds to the still images is further indicated with grey overlay on the traces. (B) The correlation between individual traces was computed. By fitting a Gaussian curve to the data, the delay times shown in Fig 5B could be determined. Plotted are means +/- SEM. Raw data can be found in S2 Data. (TIF) [file pbio.1002381.s012.tif]

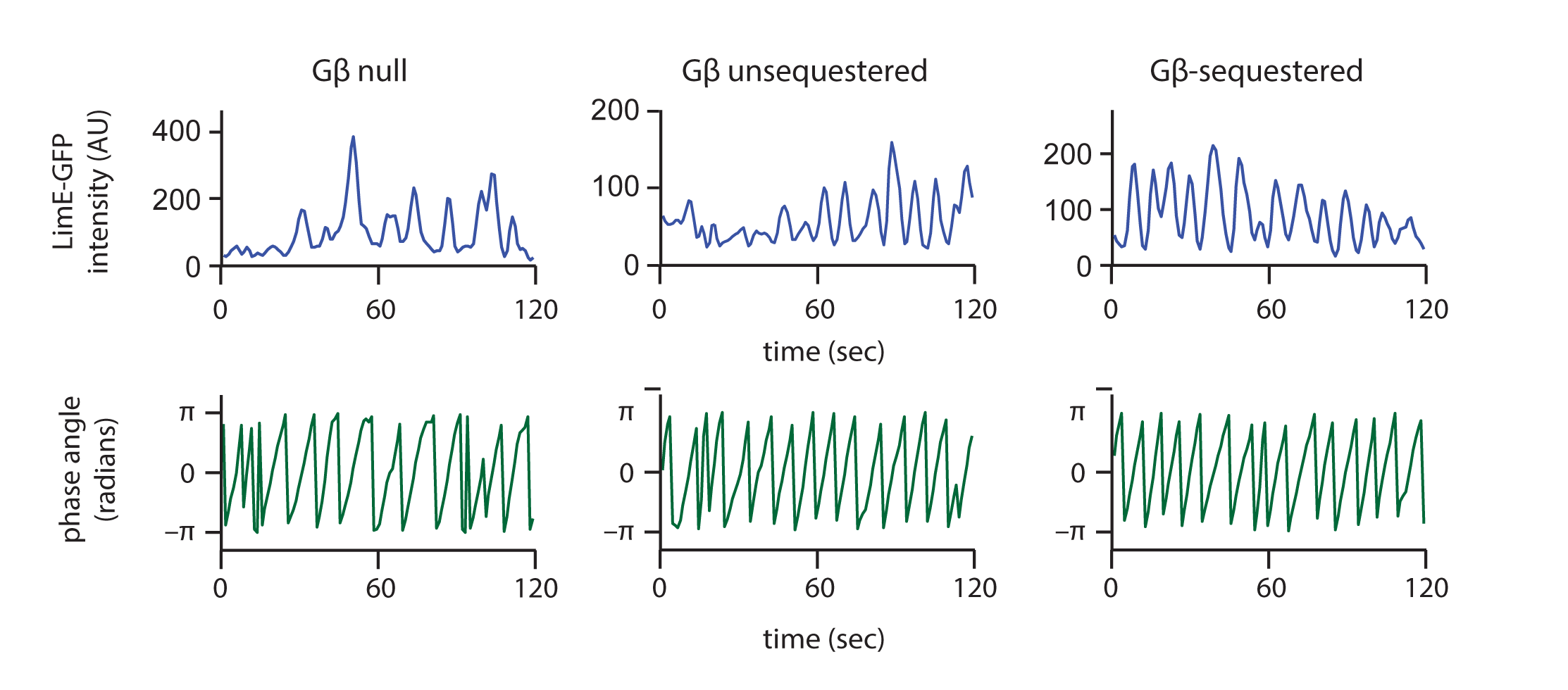

Supplement: S11 Fig — Shown are timecourses of LimE-GFP intensity from representative membrane sectors (top graphs) and the Hilbert-extracted phase information (bottom graphs) for Gβ-null, Gβ-unsequestered, and Gβ-sequestered cells (data from one cell for each condition are shown). In each case, the phase increases from -2π to 2π during each membrane LimE-GFP pulse. Raw data can be found in S2 Data. (TIF) [file pbio.1002381.s013.tif]

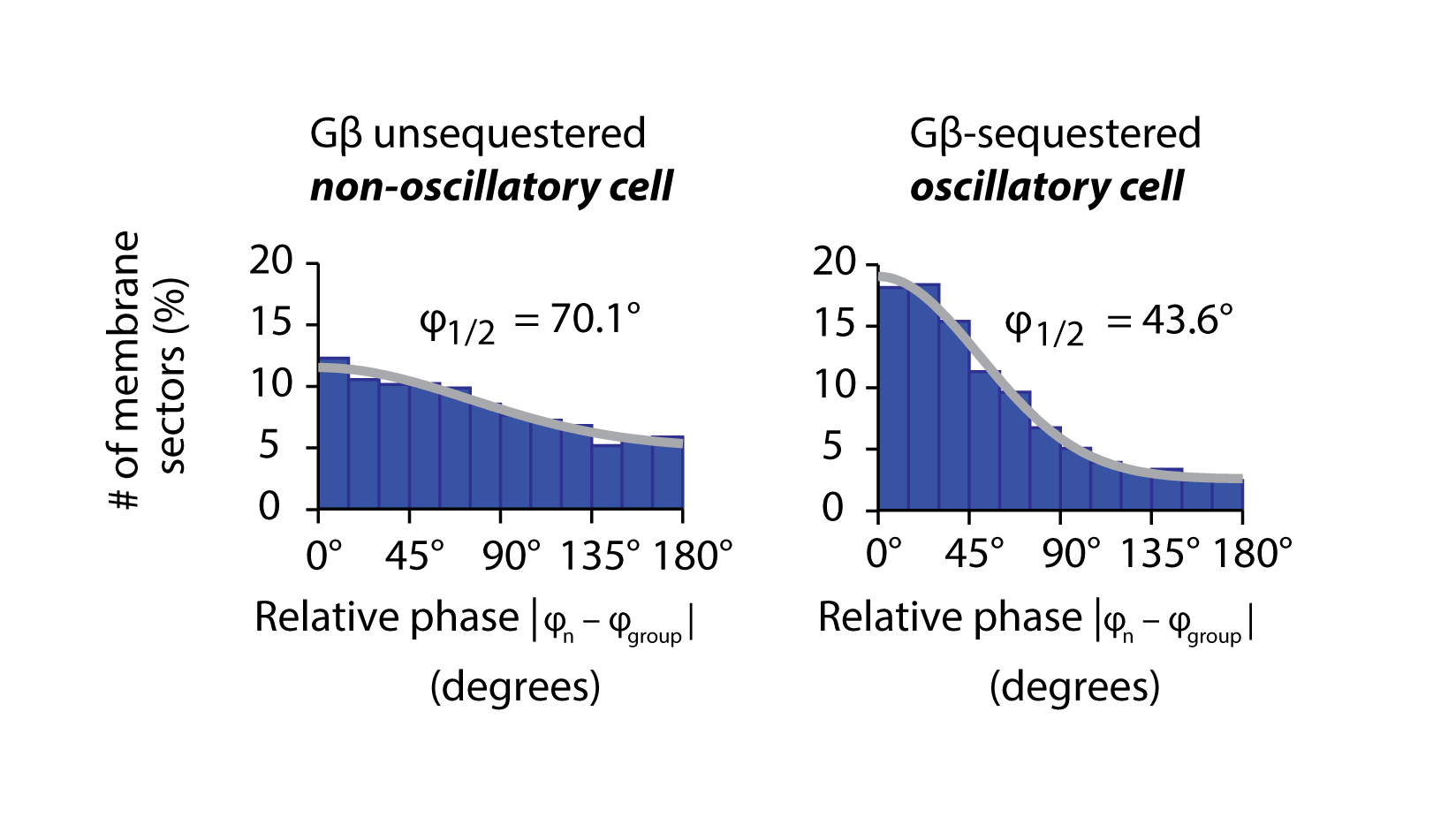

Supplement: S12 Fig — Histograms of the phase difference between each membrane sector and the “group phase” (the mean response of all membrane sectors) is shown across all sectors and timepoints for two representative cells. Cells undergoing whole-field oscillation (right panel) are more synchronous, displaying a tighter clustering around the group phase than nonoscillatory cells (left panel). It is worth noting that membrane sectors in nonoscillatory cells show weak coupling, and membrane sectors in oscillatory cells do not completely phase lock. Raw data can be found in S2 Data. (TIF) [file pbio.1002381.s014.tif]

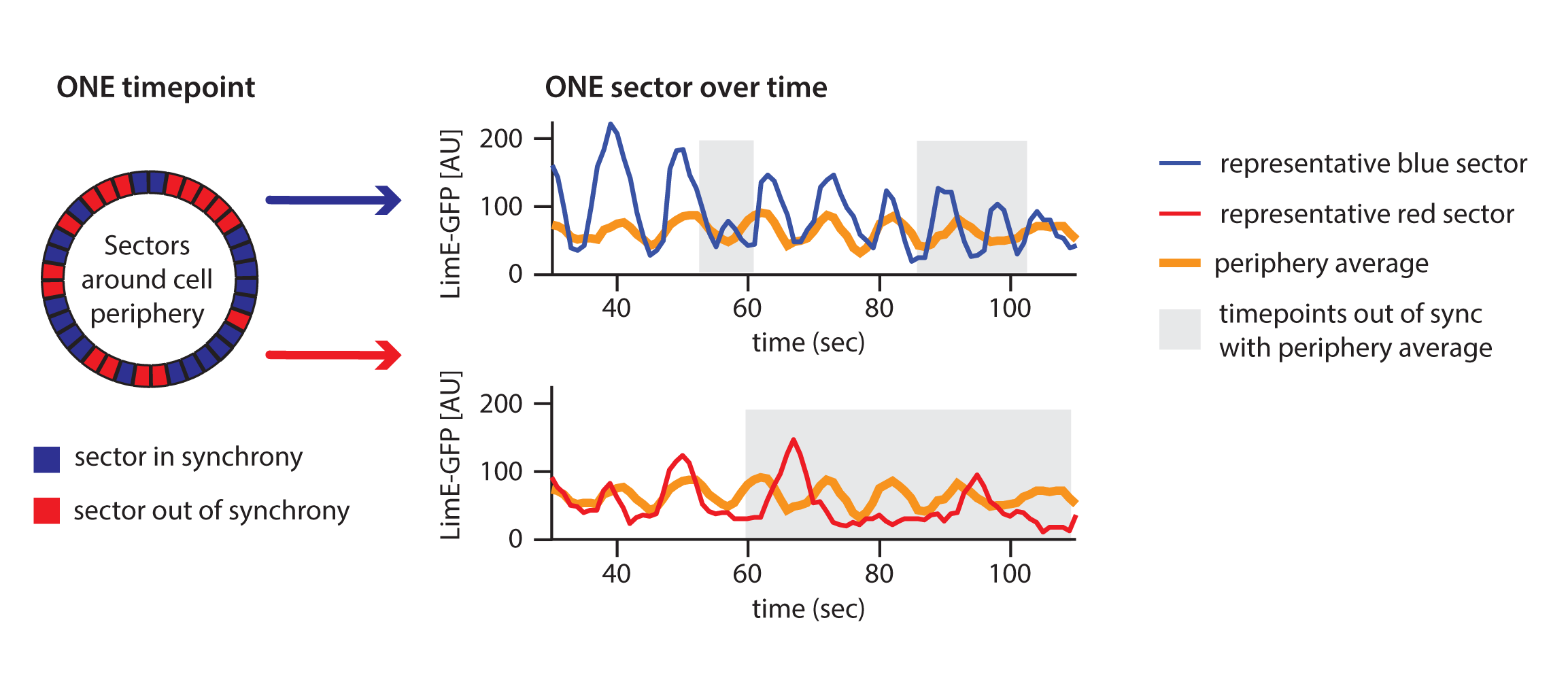

Supplement: S13 Fig — During a 50-s window, the top 25% most-synchronized membrane regions (blue) and least-synchronized membrane regions (red) of one Gβ-sequestered cell were determined (left). Following one blue sector and one red sector over time shows that, at different times, either sector can be “in sync” and “out of sync” with the periphery average. Raw data can be found in S2 Data. (TIF) [file pbio.1002381.s015.tif]

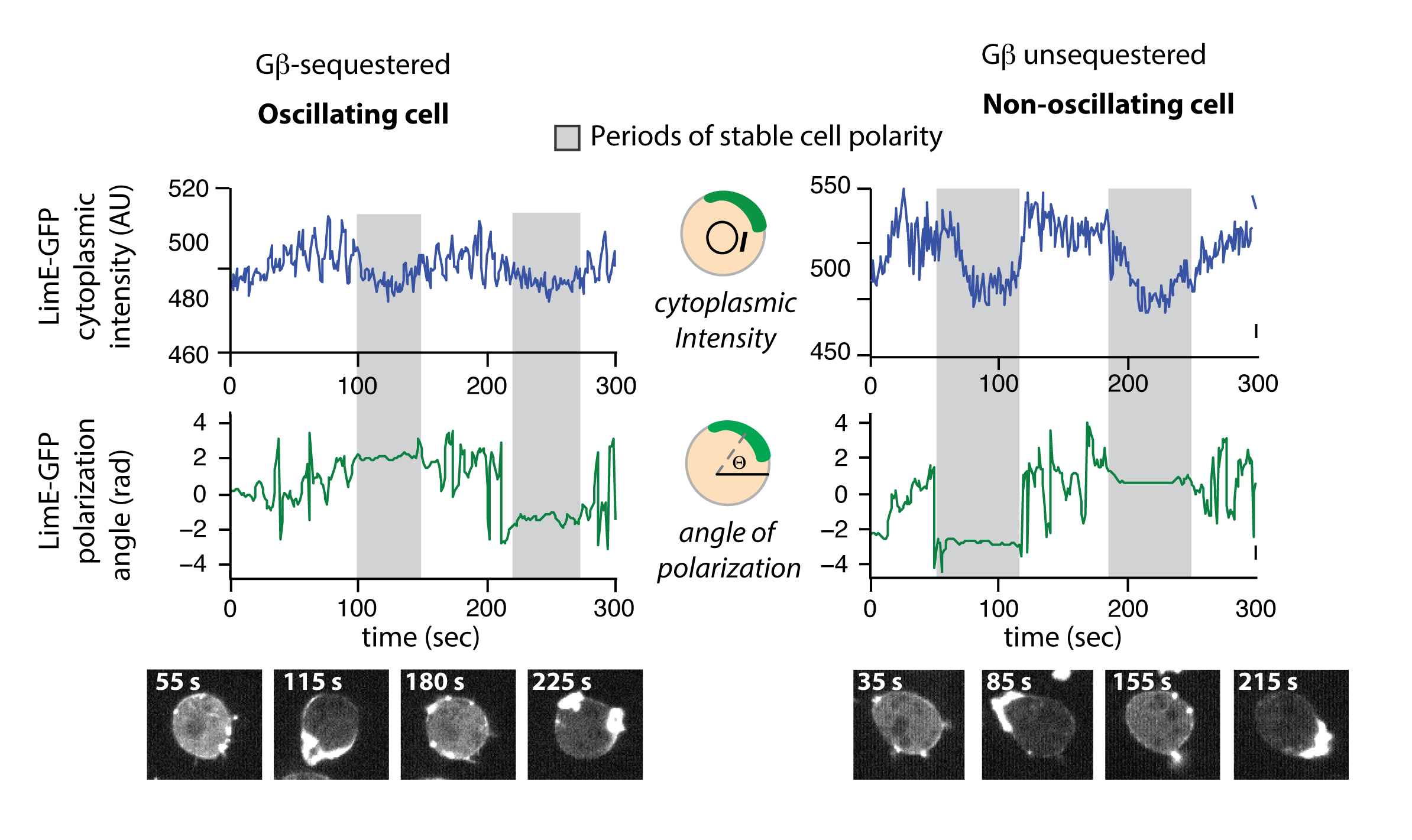

Supplement: S14 Fig — Cytoplasmic LimE-GFP intensity (upper panels) and polarization angle (lower panels) are shown for two cells expressing the Gβ-sequestration system—one Gβ-sequestered, oscillatory cell (left) and one Gβ-unsequestered, nonoscillatory cell (right). Periods of stable cell polarity are indicated in gray. Images of the cells during polarized and nonpolarized phases (below) show that a decrease in cytoplasmic intensity and the stability of pole angle are tightly correlated with the appearance of polarized regions of cortical LimE-GFP. Raw data can be found in S2 Data. (TIF) [file pbio.1002381.s016.tif]

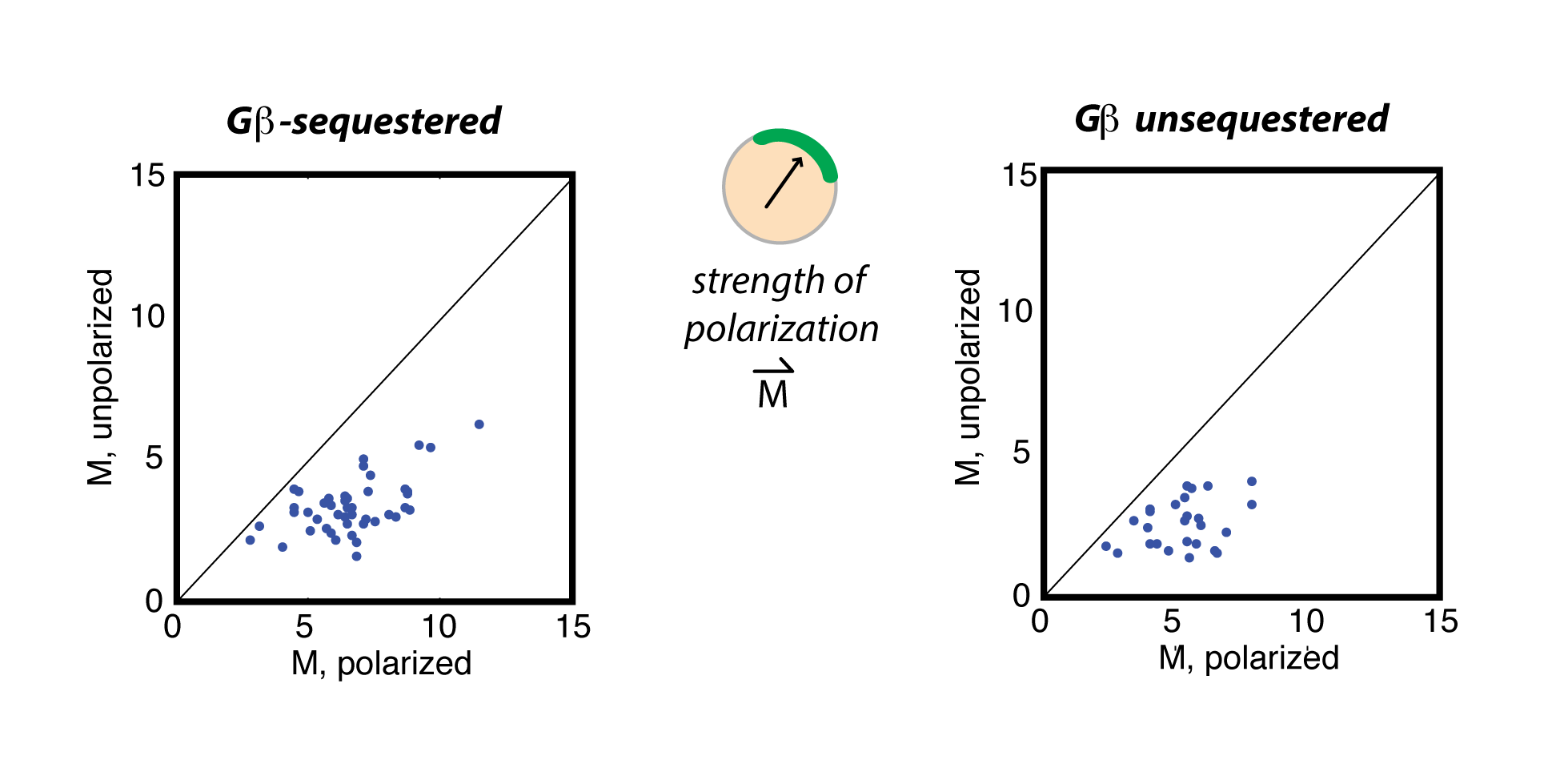

Supplement: S15 Fig — The magnitude of the vector M is plotted for individual cells during polarized and nonpolarized phases. A similar area of the diagram is occupied during polarized phases for both Gβ-sequestered and Gβ-unsequestered cells. Raw data can be found in S2 Data. (TIF) [file pbio.1002381.s017.tif]

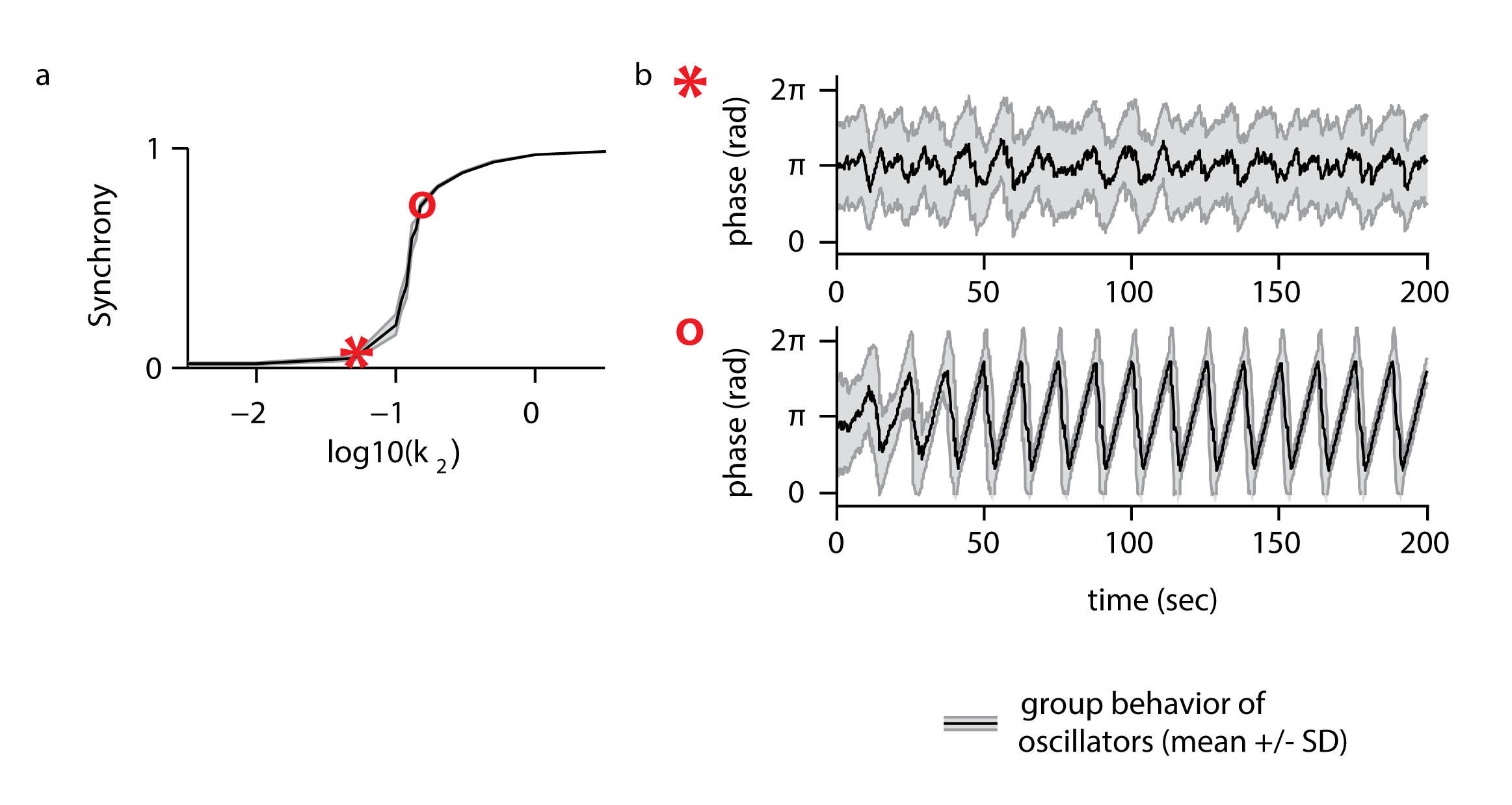

Supplement: S16 Fig — In the absence of external input, coupling in our model is only determined by parameter k 2. Varying its magnitude leads to an abrupt phase transition from weakly coupled oscillators to large-scale synchrony. This recapitulates the phenotype we observe after Gβ-sequestration. The group behavior of the oscillators for two values of k 2 is shown. Raw data can be found in S2 Data. (TIF) [file pbio.1002381.s018.tif]

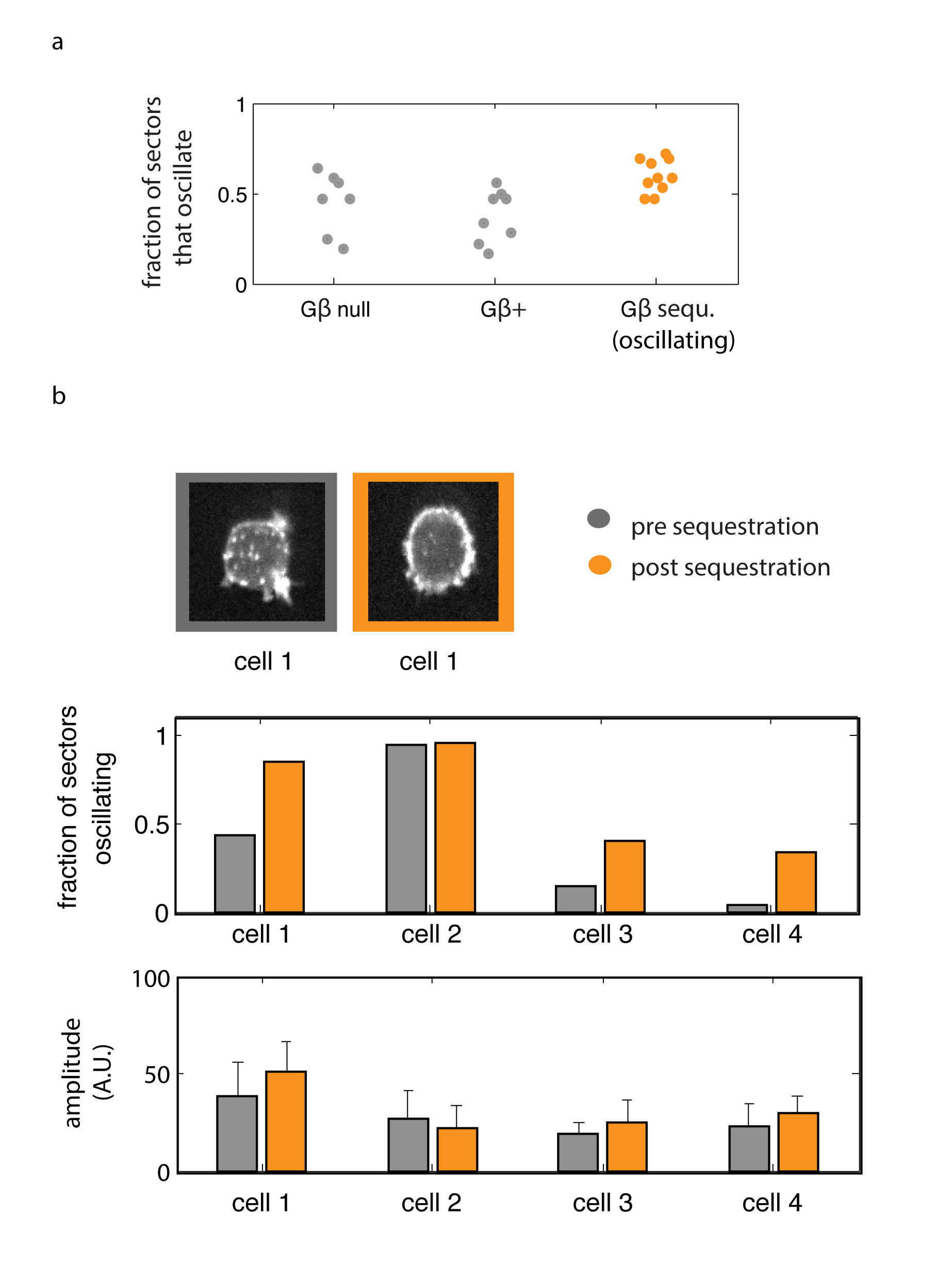

Supplement: S17 Fig — (A) Plot shows the fraction of membrane sectors containing an oscillating actin focus. We used the same dataset as in Fig 5E (Gβ-null [mutants]; Gβ+ [Gβ-unsequestered]; and Gβ-sequestered cells displaying whole-field oscillations). For whole-field oscillating cells, a higher fraction of membrane sectors contains an actin oscillator. (B) Images show a maximum intensity projection of 30 frames (1 frame/second) of a representative cell for which Gβ sequestration induces global oscillations. Cell is shown before and after the perturbation. Graphs show the fraction of membrane sectors, or the amplitude of the membrane sectors, that oscillate for four sequestered cells before and after rapamycin-mediated sequestration. Overall, amplitude is unchanged, while the fraction of oscillating sectors increases. For cell 2, all membrane sectors oscillate, although not in phase with one another, prior to Gβ sequestration. Raw data can be found in S2 Data. (TIF) [file pbio.1002381.s019.tif]

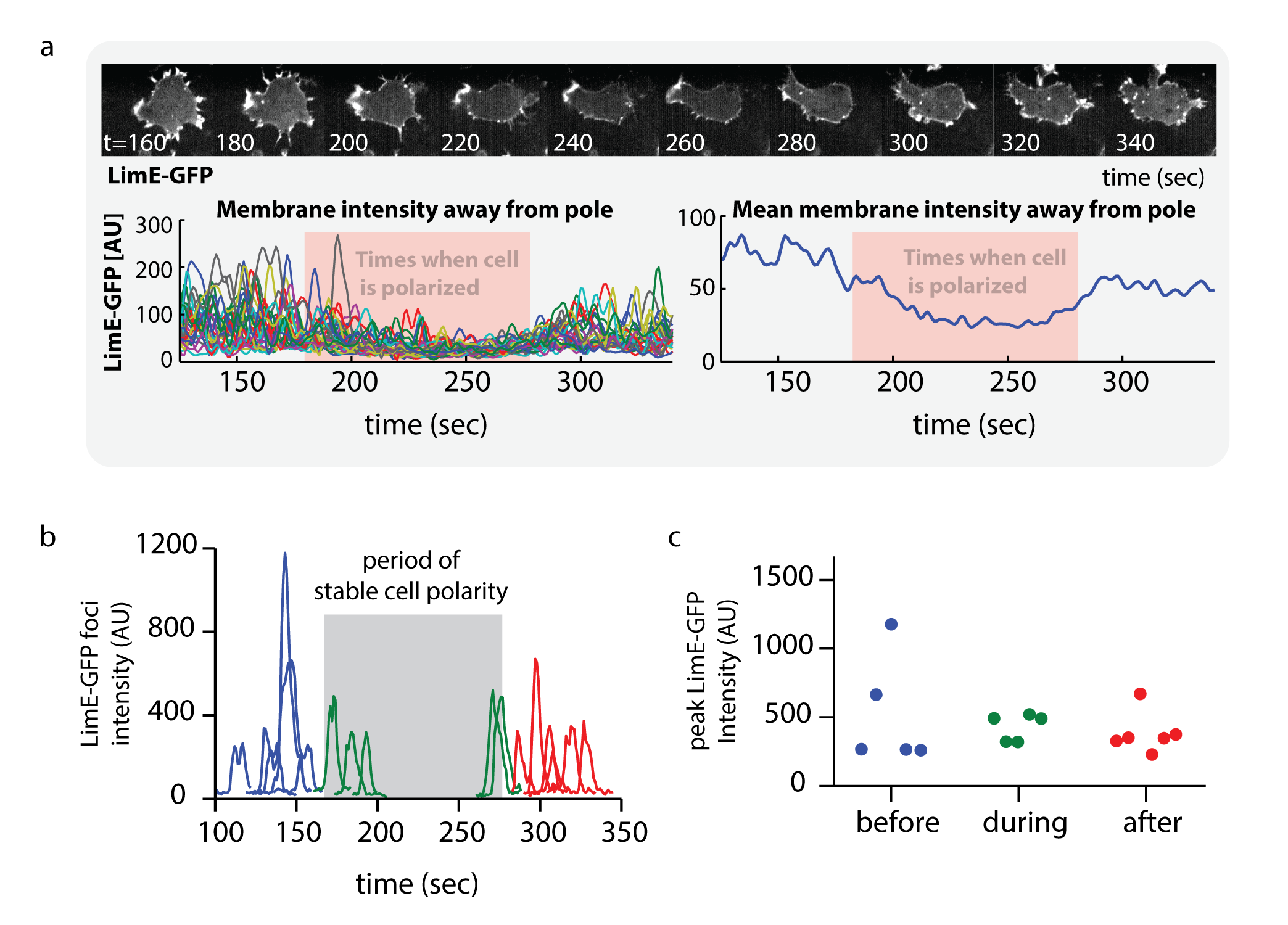

Supplement: S18 Fig — (A) The top panels show representative images of one Gβ-unsequestered cell over time as the cell forms a pole of high LimE-GFP intensity (t = 180–280 s). Bottom panels show individual intensity traces and mean intensity of all sectors not contributing to the pole. As the cell polarizes, actin foci at the periphery are lost (see also S6 Movie) (B) LimE-GFP intensity of actin foci at the periphery does not decrease because of competition with a pole. Maximum LimE-GFP intensity of individual actin structures appearing in the middle of the cell was quantified and plotted as a function of time of their appearance. Structures, most likely endocytic patches, are colored differently based on their appearance relative to a cell polarization event (gray): before polarization (blue), during polarization (green), and after polarization (red). All data are from the representative cell pictured in (A). (C) Quantitation of the peak intensity from each actin trajectory in (B). Raw data can be found in S2 Data. (TIF) [file pbio.1002381.s020.tif]

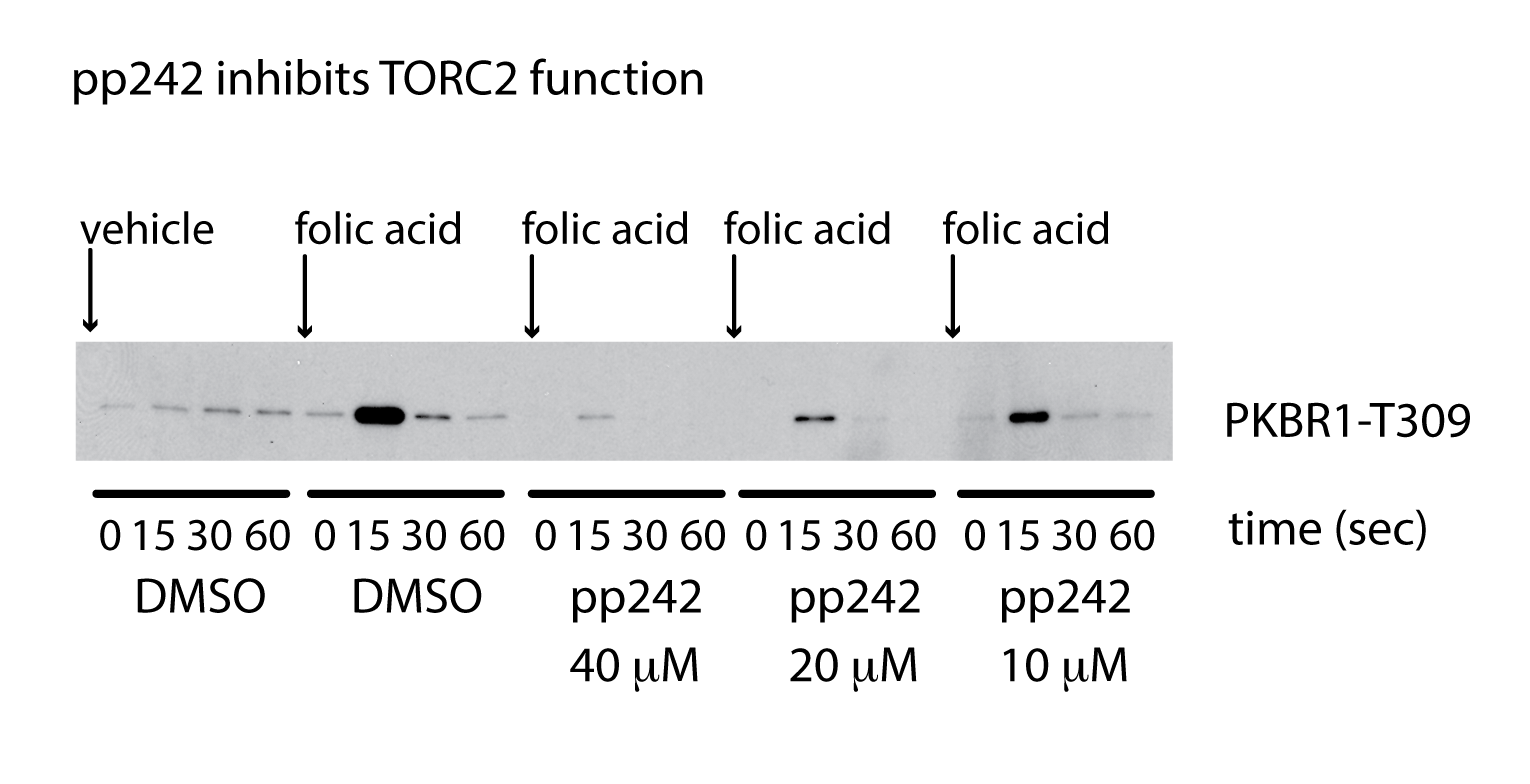

Supplement: S19 Fig — 2x10^7^ cells/ml in DB buffer were incubated with the indicated concentrations of pp242 or the DMSO solvent only. At time 0, cells were stimulated with 50 mM folic acid or buffer (vehicle control), and samples were collected and blotted for phosphorylation at T309 of PKBR1. A dose-dependent reduction in peak levels (t = 15 s) is apparent. (TIF) [file pbio.1002381.s021.tif]

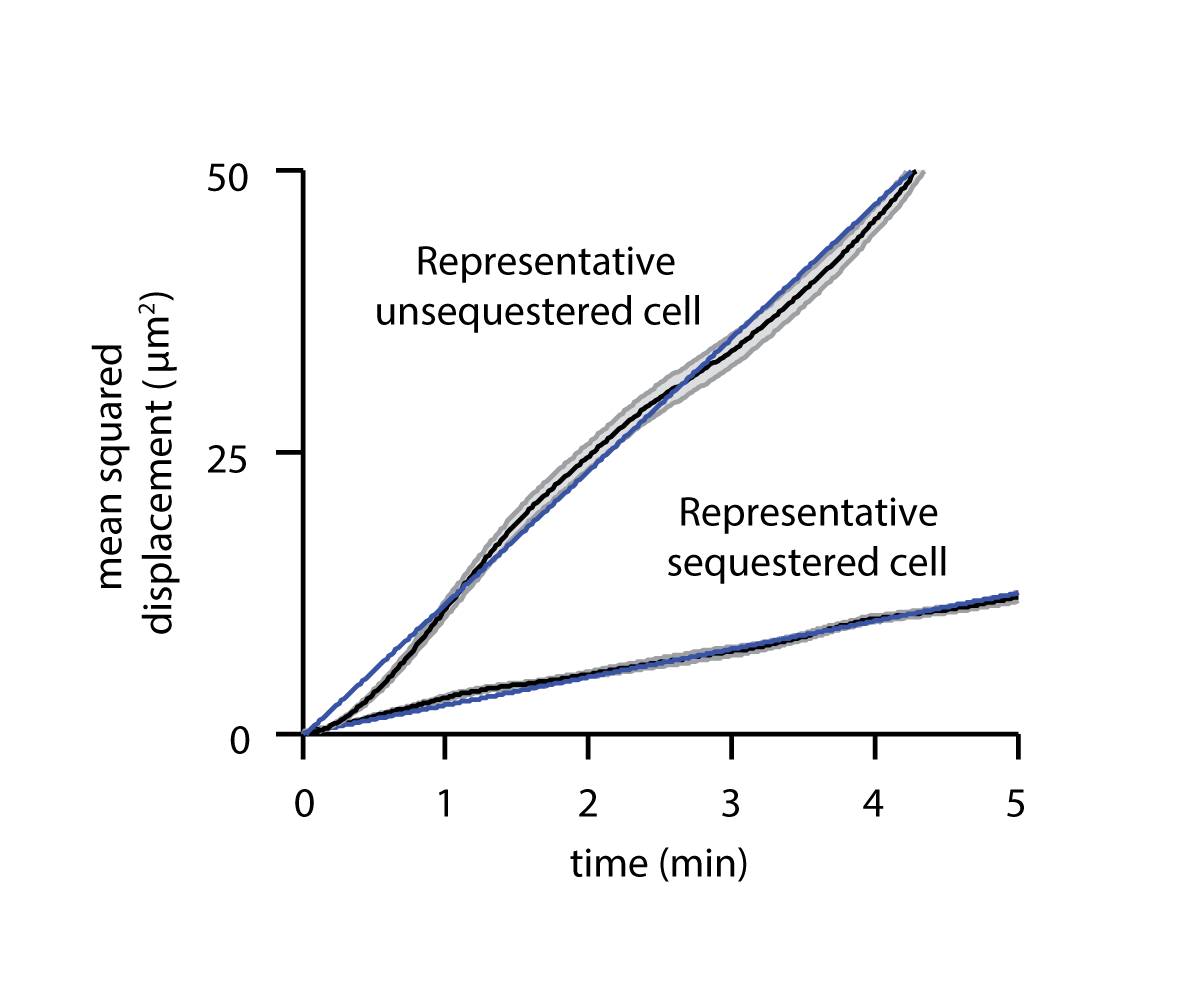

Supplement: S20 Fig — Plot of the mean squared displacement (mean +/- SEM) as a function of time for two representative cells, one for which Gβ has been sequestered and one for which Gβ has not been sequestered. Cells were automatically tracked by their centroid from thresholded fluorescent images taken each second (same data from which numbers for Fig 7C and 7D were extracted). Each cell’s mean squared displacement was calculated from all trajectories during a 5-min interval of a 10-min movie. Best-fit line (proportional to the estimated diffusion coefficient) is shown in blue. Raw data can be found in S2 Data. (TIF) [file pbio.1002381.s022.tif]

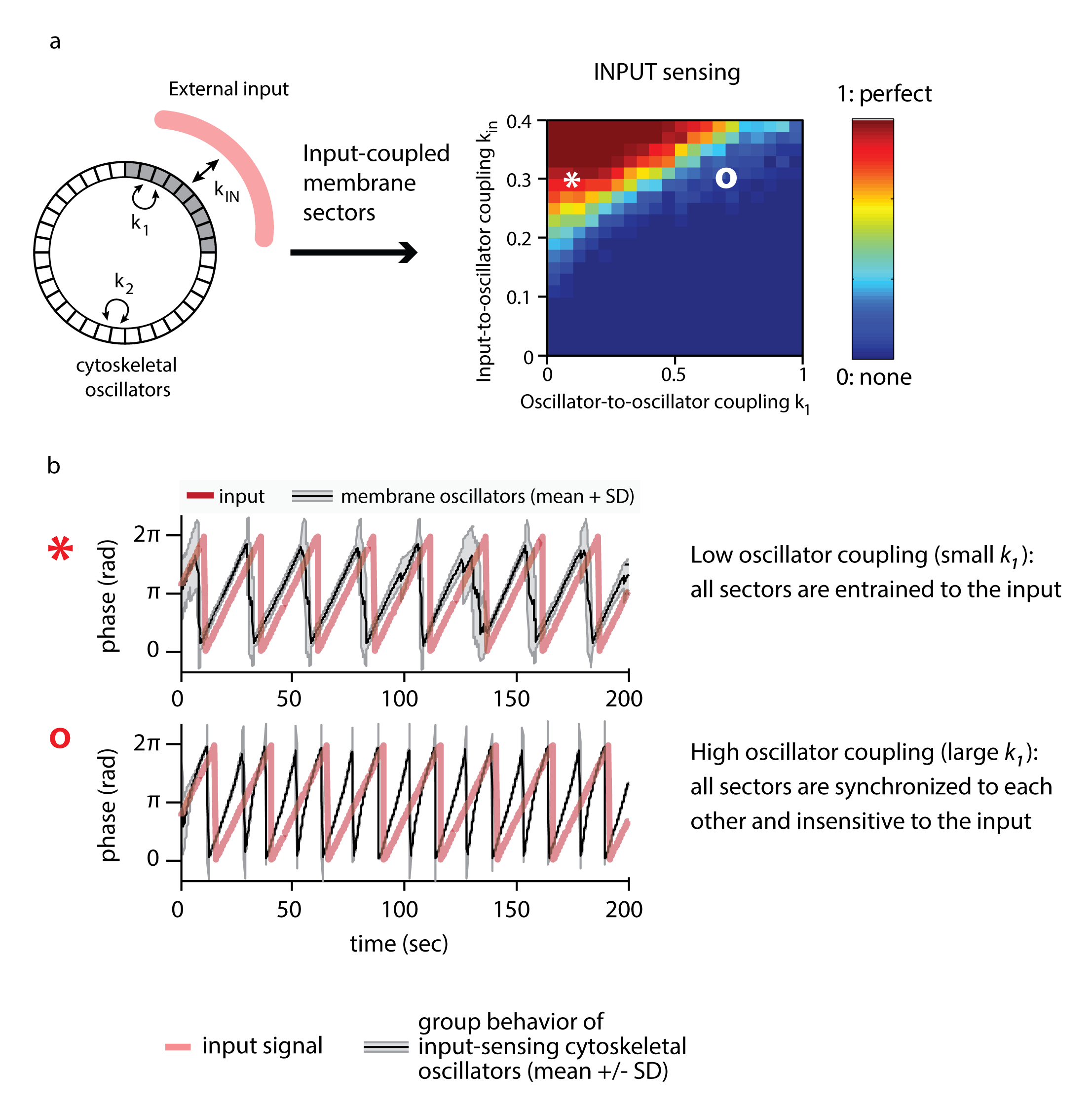

Supplement: S21 Fig — (A) We kept k 2 at a fixed, low value (0.1) and addressed how oscillator-to-oscillator coupling in an area experiencing an input (k 1) affected sensing of inputs (k IN). High values of k IN lead to perfect synchronization of input and oscillator dynamics (input sensing). Increasing k 1 only decreases the extent of synchronization (see heat map and graph). (B) Traces of indicated, representative areas of the heat map in (A) are shown. Raw data can be found in S2 Data. (TIF) [file pbio.1002381.s023.tif]
